# Mycobacterial infections and recurrent osteomyelitis in a patient with an autosomal dominant mutation in IFN-gamma receptor 1 with overexpression of the receptor

**DOI:** 10.1016/j.ijid.2026.109104

**Published:** 2026-09-06

**Authors:** Pamela Rivero-García, Luis Martínez-Robles, Yolanda González, Laura Ventura-Ayala, Eric Ochoa-Hein, Armando Gamboa-Domínguez, Jean-Laurent Casanova, Stephanie Boisson-Dupuis, Sigifredo Pedraza-Sánchez

**Affiliations:** 1 Department of Genetics, Instituto Nacional de Ciencias Médicas y Nutrición Salvador Zubirán (INCMNSZ), Mexico City 14080, Mexico; 2 Unit of Biochemistry, Instituto Nacional de Ciencias Médicas y Nutrición Salvador Zubirán (INCMNSZ), Mexico City 14080, Mexico; 3 Department of Research in Microbiology, Instituto Nacional de Enfermedades Respiratorias Ismael Cosío Villegas (INER), Mexico City 14080 Mexico.; 4 Department of Hospital Epidemiology, Instituto Nacional de Ciencias Médicas y Nutrición Salvador Zubirán (INCMNSZ), Mexico City 14080, Mexico; 5 Department of Pathology, Instituto Nacional de Ciencias Médicas y Nutrición Salvador Zubirán (INCMNSZ), Mexico City 14080, Mexico; 6 Current affiliation: Tecnológico Nacional de México / Instituto Tecnológico Superior de Tierra Blanca, Tierra Blanca, Veracruz, 95180, México.; 7 St. Giles Laboratory of Human Genetics of Infectious Diseases, Rockefeller Branch, Rockefeller University, New York, NY, USA.; 8 Laboratory of Human Genetics of Infectious Diseases, Necker Branch, INSERM UMR 1163, Necker Hospital for Sick Children, Paris, France.; 9 Paris Cité University, Imagine Institute, Paris, France.; 10. Department of Pediatrics, Necker Hospital for Sick Children, Paris, France.; 11 Howard Hughes Medical Institute, New York, NY, 10065, USA.

## Abstract

Mutations in genes controlling IFN-γ production or signaling underlie Mendelian susceptibility to mycobacterial disease (MSMD) and predispose affected individuals to mycobacterial and other infections. Autosomal dominant (AD) mutations in exon VI of *IFNGR1*, which encodes interferon-γ receptor 1 (IFN-γR1), have been associated with multifocal osteomyelitis. We report a Mexican male patient whose clinical course began with severe BCGitis at 2 years of age, followed by tuberculous lymphadenitis at age 3, disseminated tuberculosis with hepatitis at age 10, and disseminated *Mycobacterium bovis* infection with vertebral osteomyelitis at age 19. Subsequent manifestations included HPV-associated warts at age 21, pneumonia and vertebral osteomyelitis associated with *M. colombiense* at age 25, mesenteric fibrosis, hepatic abscesses, vertebral osteomyelitis associated with *M. avium* at age 28, and pneumonia associated with *M. fortuitum* at age 30. The patient responded to prolonged antimycobacterial therapy. Functional studies demonstrated deficient IL-12p40 production following BCG plus IFN-γ stimulation, impaired Stat-1 phosphorylation in response to IFN-γ, and increased cell-surface expression of CD119/IFN-γR1. Sanger sequencing identified the heterozygous *IFNGR1* c.805delT (p.Tyr269Ilefs*8) variant in the patient but not in his parents, consistent with a *de novo* event. This truncating variant leads to cell-surface accumulation of a defective IFN-γR1 and exerts a dominant-negative effect on IFN-γ signaling. This case, together with our literature review, supports an association between impaired IFN-γ signaling due to AD *IFNGR1* variants and susceptibility to recurrent infectious osteomyelitis.

## Introduction

In humans, different Inborn Errors of Immunity (IEI) predispose individuals to Mycobacterial infection and disease. Mendelian susceptibility to mycobacterial diseases (MSMD) comprises distinct IEIs associated with the IL-12 -IL23 / IFN-γ axis. Mutations in genes controlling the IFN-γ production (*IFNG*, *IL12B*, *IL12RB1*, *IL12RB2*, *IL23R*, *TBX21*, *TYK2, MCTS1, ISG15*), response (*IFNGR1*, *IFNGR2*, *STAT1*, *JAK1, CYBB, USP18*), or both (*IRF1, IRF8, SPPL2A, NEMO*) may cause predisposition to infections, mainly by *Mycobacterium*, *Salmonella*, *Candida* or virus infections, of variable severity [1; 2]. Infections caused by other microorganisms have also been reported in individuals with these IEIs. Mutations in *IL12RB1*, which encodes the IL-12 receptor β1 subunit, as well as *IFNGR1* and *IFNGR2*, which encode for the IFN-γ receptor chains 1 and 2, respectively, account for 78% of MSMD cases worldwide [3] .

The gene *IFNGR1* has seven exons [4; 5] and more than 40 different mutations across all exons have been reported to date [6] . Autosomal recessive (AR) mutations occur in exons I-V and VII and can result in either a complete or partial defect, leading to null or low receptor expression and severe disease phenotypes. In contrast, autosomal dominant (AD) mutations occur in exon VI, which encodes the transmembrane domain of the receptor (see Supplementary Table S1). These AD mutations or pathogenic variants produce partial defects characterized by high protein expression and accumulation at the cell membrane and are associated with a lower mortality (10%) before age 20, compared to 80% in patients with AR mutations [7]. Atypical mycobacterial infections are the most common among patients with mutations in *IFNGR1*, mostly *Mycobacterium avium* Complex (MAC).

### The clinical case

We studied a male patient from Mexico, born to non-consanguineous parents, whose clinical long story of infections began at 2 years of age with an axillary abscess secondary to BCG vaccination. This was diagnosed as severe BCGitis and managed successfully with a three-month course of rifampin. A year later, he developed submandibular and jugular lymphadenopathy accompanied by nodular facial skin lesions. Despite inconclusive histopathology, he was diagnosed clinically with tuberculous lymphadenitis and underwent a nine-month regimen of isoniazid, rifampin, clarithromycin, and ciprofloxacin, resulting in clinical improvement.

At age 10, the patient presented with persistent fever and an abdominal mass. Imaging revealed hepatic nodules, and laparotomy confirmed caseating granulomatous hepatitis with a positive PCR for *Mycobacterium tuberculosis*. Treatment involved a 12-month regimen of isoniazid, rifampin, amikacin, and ethambutol. However, the therapy was complicated by drug-induced bicytopenia, necessitating multiple blood transfusions. At age 17, he developed a right elbow fissure, which was managed conservatively.

At the age of 19, the patient was diagnosed with disseminated infection by *Mycobacterium bovis* through gastric fluid culture and hepatic biopsy findings. The patient required intensive care management for septic shock. Thoracic CT showed bilateral nodular pulmonary lesions (Supplementary Figure S1A). He also developed multifocal vertebral osteomyelitis (Pott’s disease), demonstrated by CT (Figure 1A). Bone biopsy showed abundant acid-fast bacilli within the foamy cytoplasm of histiocytes (Figure 1B), together with osteoblast rimming and a paratrabecular cluster of epithelioid histiocytes (Supplementary Figure S1B), confirming bona fide mycobacterial bone infection. He received a prolonged multidrug regimen consisting of moxifloxacin, isoniazid, rifampin, and ethambutol for 18 months, leading to partial resolution of his symptoms and imaging abnormalities. During this period, systemic candidemia caused by *C. parapsilosis*, sepsis caused by *Pseudomonas* and kidney infection caused by *E. coli* also developed and were managed accordingly. The patient was subsequently discharged and continued fluconazole for 6 months and anti-mycobacterial drugs for 18 months.

Given the recurrent mycobacterial infections, an IEI related to MSMD was suspected. Laboratory tests for evaluation of the IFN-γ signaling pathway found a deficient production of IL-12p40 upon BCG + IFN-γ co-stimulation (Supplementary Figure S1C), suggesting a possible defect in the IFN-γ receptor or in Stat-1. Impaired Stat-1 phosphorylation in response to IFN-γ was demonstrated by flow cytometry (Figure 1C and Supplementary Figure S2) and Western blot (Supplementary Figures S1D and S5). We also found abnormally high expression of IFN-γR1 or CD119 in cells from the patient, but not in other family members, suggesting an altered receptor (Figure 1D, and Supplementary Figures S3 and S4). Sequencing of the *IFNGR1* gene revealed a pathogenic variant c.805delT (p.Tyr269Ilefs*8) in the patient but not in other family members, suggesting a *de novo* event (Supplementary Figures S1E and S6). The variant was classified as pathogenic according to ACMG/AMP criteria. These findings are compatible with MSMD. The same *IFNGR1* c.805delT variant was previously reported [8] . The main clinical and functional findings of both patients are compared in Supplementary Table S2

Between 2017 and 2023, the patient experienced recurrent infections with other mycobacteria, including *M. colombiense, M. avium and M. fortuitum*. These episodes presented with variable respiratory symptoms, soft tissue infections, and systemic inflammatory responses. Each episode required targeted anti-mycobacterial regimens based on culture sensitivities and biopsy findings. Notably, infections with *M. colombiense* and *M. avium* were complicated by abscess formation in the liver and mesentery, osteomyelitis and fibrosis, requiring surgical interventions and prolonged treatment courses.

Currently, the patient is clinically stable and still under clarithromycin and doxycycline treatment for *M. fortuitum*. He remains asymptomatic, with negative microbiological tests and is under regular follow-up. A timeline of the clinical case is presented in Supplementary Figure S7**.**

The c.805delT mutation is located in exon VI of *IFNGR1*, which encodes for the transmembrane domain of the receptor. This region is crucial for receptor recycling, and mutations therein cause accumulation of defective incomplete IFN-γR1 chains lacking intracytoplasmic domain. The functional IFN-γ receptor is a tetramer composed of two IFN-γR1 and two IFN-γR2 peptides, displayed on the membrane of most nucleated cells [6; 9; 10] . Accumulation of mutant IFN-γR1 disrupts this 2:2 normal stoichiometry, and a non-functional receptor tetramer that includes incomplete R1 (without intracytoplasmic domain) is displayed on the membrane. This impairs Stat-1 phosphorylation and downstream cytokine transcription needed for phagocyte activation and mycobacterial clearance (Supplementary Figure S8**)**. Other variants in the *IFNGR1* exon VI have also been associated with high expression of IFN-γR1 chain and atypical mycobacterial infections.[11; 12; 13; 14]

Mutations located in distinct exons of *IFNGR1* have different genetic and receptor expression profiles (Supplementary Table S1**,**). Patients with AR mutations lack IFN-γ receptor expression or express a defective IFN-γ receptor chain 1, affecting IFN-γ binding, signaling, or both. Patients with AR mutations suffer mainly of early-onset, severe and persistent MAC infections, with high mortality rate. In contrast, patients with AD mutations in *IFNGR1* are all located in exon VI, overexpress the IFN-γR1 chain, leading to defective signaling. Although they also present with recurrent mycobacterial infections, they generally have better outcomes with antimicrobial therapy.

There is an autoinflammatory bone disorder called chronic recurrent multifocal osteomyelitis (CRMO), which is not caused by infection, but is associated with dysregulation of pro-and anti-inflammatory cytokine production [15] CRMO occurs predominantly in children and adolescents and may be accompanied by inflammatory manifestations such as synovitis, acne and pustulosis. These features were not observed in our patient, whose episodes of osteomyelitis occurred in the context of microbiologically documented infections, supporting an infectious rather than autoinflammatory aetiology.

Infectious osteomyelitis appears to be more frequent in patients with AD *IFNGR1* mutations than in those with AR variants, most commonly associated with *Mycobacterium* species, although fungal and bacterial pathogens have also been reported, often with multifocal involvement (Supplementary Table S3). Consistent with this genotype-phenotype association, our literature review showed osteomyelitis in 39/51 (76.5%) patients with AD *IFNGR1* variants compared with 11/25 (44.0%) patients with AR variants. Recurrent and/or multifocal osteomyelitis showed an even greater difference, occurring in 37/51 (72.5%) and 8/25 (32.0%) patients, respectively (Supplementary Table S4). Similarly, Dorman et al. reported that osteomyelitis occurred in 22/28 patients (78%) with AD mutations, all carrying the c.819_822del (p.Asn274Hisfs*2) variant, also known as c.818del4. Remarkably, in one-third of those patients, multifocal Mycobacterial bone infection was the only clinical manifestation [7] . In contrast, only 5 out of 38 patients with AR mutations had osteomyelitis, and none had it as the sole infection. This unexpected lower frequency of osteomyelitis in patients with AR mutations in *IFNGR1* compared to AD mutations may reflect early mortality from severe infections in patients with AR mutations.

The physiological bone homeostasis depends on the balance between new-bone production by osteoblasts and bone resorption by osteoclasts. IFN-y normally inhibits osteoclast differentiation; accordingly, impaired IFN-γ signaling in patients with AD *IFNGR1* and *STAT1* mutations has been associated with enhanced osteoclastogenesis and increased osteoclast numbers [16] . This mechanism may contribute to the increased susceptibility to multifocal osteomyelitis observed in these patients. However, bone involvement is likely multifactorial, and chronic inflammation, recurrent mycobacterial burden, and cumulative structural damage from previous infectious episodes may also contribute to recurrent and multifocal osteomyelitis.

Osteomyelitis has also been described in patients with anti- IFN-gamma antibodies (AIGA) [17] (Supplementary Table S4). AIGA were not assessed in our patient; however, AIGA-associated immunodeficiency was considered unlikely because it is typically an acquired condition presenting in adulthood and at the best of our knowledge, it has been reported only in individuals from East and Southeast Asia. In contrast, our patient developed his first infectious manifestations at 2 years of age following BCG vaccination and carries a pathogenic *IFNGR1* variant with a concordant functional defect in IFN-γ signaling.

### Conclusion

Although the c.805delT variant in *IFNGR1* gene has been previously reported, the present case provides a detailed longitudinal characterization from early childhood to adulthood, including recurrent infections caused by multiple mycobacterial species, repeated multifocal osteomyelitis, long-term treatment outcomes, and functional characterization of the IFN-γR1 defect. Therefore, our study expands our knowledge on this kind of IEI.

In conclusion, disseminated and recurrent mycobacterial infections, along with multifocal osteomyelitis may indicate an IEI due to an AD *IFNGR1* mutation, as in the present case, or other defects in the IFN-γ signaling pathway that warrant further investigation and diagnosis.

## Supplementary Material

REFERENCES FOR SUPPLEMENTARY TABLES

Supplementary Figure S2 **Supplementary Figures 2, 3 and 4** and their figure legends are included in Word Supplementary file “Supplementary figures 2,3 and 4”, which includes also additional methods for flow cytometry and western blot.

Supplementary Figure S3

Supplementary Figure S4

Supplementary Figure S1 **Supplementary Figure S1. A** Thorax CT showing nodular lesions in both lungs. **B** Osteoblast rimming a bone spicule (arrowhead) with a paratrabecular cluster of epithelioid histiocytes (arrow) admixed with myeloid precursors **C** IL-12 production by diluted whole blood, from healthy donors (n = 10, blue points) and patient (red square), stimulated as indicated and assessed by ELISA in 48h supernatants. NS, Non-stimulated **D** p-Stat-1 detected by Western Blot in whole cell lysates from EBV-LCL from patient, his mother and a healthy control, stimulated by 20 minutes as indicated **E** Schematic representation of the IFN-γR1 structure and the position of the mutation. EC, TM and IC, extracellular, transmembrane and intracellular domains, respectively. The family pedigree shows that the patient was the only carrier of pathogenic variant in *IFNGR1*.

Supplementary Figure S5 **Supplementary Figure S5. Non-edited Western Blot for Stat-1 and p-Stat-1 from whole cell lysates from patients and controls.** EBV-LCL from a healthy positive control, negative control (from a second patient with a distinct *IFNGR1* heterozygous variant), the patient’s mother and the patient, were incubated during 20 minutes with no stimulus or 100 or 1000 IU IFN-gamma, and reaction was stopped with paraformaldehyde. Whole cell lysates were obtained and Total Stat-1 and phospho -Stat-1 (p-Stat-1) were detected by western blot, as previously published (Pedraza-Sanchez, Lezana JL, Gonzalez Y et al, 2017. Front Immunol. 8:1651). The order of the lines are the same for all the donors, from left to right: unstimulated, 100 IU IFN-gamma and 1000 IU IFN-gamma. The figure shows the same film developed with distinct exposure times. The experiment was repeated three times.

Supplementary Methodology

Supplementary Figure S8 **Supplementary Figure S8.** Model of mutant IFN-γ Receptor 1 and mechanism of dominant negative effect. In the left part of the figure, WT IFN-γR1 and WT IFN-γR2 form a tetrameric receptor that binds IFN-γ and induces normal downstream signaling required for phagocyte activation. At the right, the c.805-delT mutation prevents complete transcription of the polypeptide and disrupts the recycling motif of IFN-γR1 (red star), resulting in accumulation of a truncated receptor at the cell membrane. These mutant receptors can still dimerize with WT IFN-γR2 and can bind IFN-γ, but are unable to signal, thereby impairing IFN-γ– mediated cellular responses.

Supplementary Figure S7 **Supplementary Figure S7.** Timeline of the patient presented in the clinical case. The main clinical symptoms, proven infections and treatments are shown.

Supplementary Table S1

Supplementary Table S2

Supplementary Table S4

Supplementary Table S3

Supplementary Figure S6 Supplementary Figure S6. Sanger sequencing of the *IFNGR1* c.805delT (p.Tyr269Ilefs*8) variant. Genomic DNA from EBV-transformed lymphoblastoid cell lines (EBV-LCLs) was extracted using the phenol-chloroform method. The seven exons of *IFNGR1* and the 22 exons of *STAT1* were amplified by PCR using specific forward and reverse primers. PCR products were recovered from agarose gels and sequenced using the BigDye Terminator kit on an ABI Prism 377 Sequence Analyzer (PerkinElmer Systems). Sequences were compared with the corresponding reference sequences using SeqMan Pro (DNASTAR). Representative electropherograms show the heterozygous *IFNGR1* c.805delT (p.Tyr269Ilefs*8) variant in the proband and its absence in both parents, supporting a *de novo* occurrence.

## Figures and Tables

**Figure 1.**
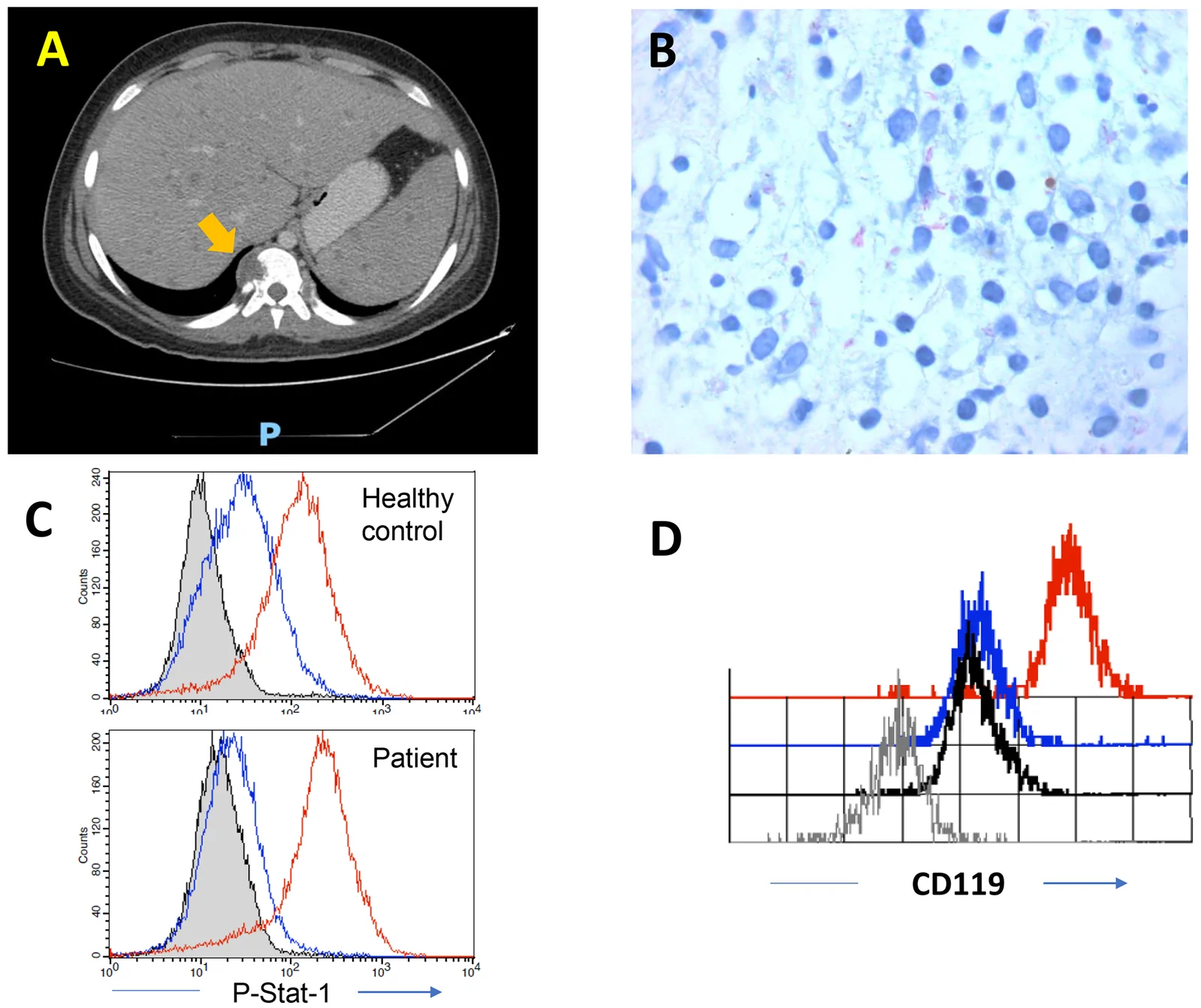
**A** Abdominal CT of patient with hepatomegaly and splenomegaly with multiple nodular foci. Osteomyelitis is shown in the vertebra (arrow). **B** Bone biopsy of patient: In the foamy cytoplasm of histiocytes, abundant acid-fast positive bacilli are observed (Ziehl-Neelsen X1000). **C** p-Stat-1 detected by intracellular Flow cytometry; EBV-LCL were stimulated or not (gray) during 20 min with IFN-γ (blue) or IFN-α (red), and p-Stat1 was detected intracellularly. **D** Expression of IFN-γ Receptor 1 on CD14+ blood monocytes (gray, isotype control, blue patient’s father, black healthy control and red, patient’s cells.

## Highlights:

- Mutations affecting IFN-γ pathway cause Mendelian Susceptibility to Mycobacterial Diseases MSMD
- Persons with MSMD are susceptible to mycobacterial, fungal, viral and other bacterial infections
- We describe an adult patient with a mutation in *IFNGR1* gene, osteomyelitis and multiple infections
- Persons with altered response to IFN-γ are susceptible to osteomyelitis and severe infections.
