# Supplementary material for: Mycobacterial infections and recurrent osteomyelitis in a patient with an autosomal dominant mutation in IFN-gamma receptor 1 with overexpression of the receptor
Source: Int J Infect Dis. Author manuscript; Available in PMC 2026 Nov 5. (PMC13739071; doi:10.1016/j.ijid.2026.109104)
Supplement: Supplementary file 1 [file NIHMS2218719-supplement-REFERENCES_FOR_SUPPLEMENTARY_TABLES.pdf]

Mycobacterial infections and recurrent osteomyelitis in a patient with an autosomal dominant mutation in IFN-gamma receptor 1 with overexpression of the receptor.

Pamela Rivero-García[1], Luis Martínez-Robles[2, 6], Yolanda González[3], Laura Ventura-Ayala[2], Eric Ochoa-Hein[4], Armando Gamboa-Domínguez[5], Jean-Laurent Casanova[7-11], Stephanie Boisson-Dupuis[7-9], Sigifredo Pedraza-Sánchez[2, #].

REFERENCES FOR SUPPLEMENTARY TABLES.

SUPPLEMENTARY TABLE 1.

[1] Jouanguy E, Dupuis S, Pallier A, Doffinger R, Fondaneche MC, Fieschi C, et al. In a novel form of IFN-gamma receptor 1 deficiency, cell surface receptors fail to bind IFN-gamma. J Clin Invest. 2000 May;105(10):1429-36.

[2] Rosenzweig S, Dorman SE, Roesler J, Palacios J, Zelazko M, Holland SM. 561del4 defines a novel small deletion hotspot in the interferon-gamma receptor 1 chain. Clin Immunol. 2002 Jan;102(1):25-7.

[3] Jouanguy E, Altare F, Lamhamedi S, Revy P, Emile JF, Newport M, et al. Interferon-gamma-receptor deficiency in an infant with fatal bacille Calmette-Guerin infection. N Engl J Med. 1996 Dec 26;335(26):1956-61.

[4] Chantrain CF, Bruwier A, Brichard B, Largent V, Chapgier A, Feinberg J, et al. Successful hematopoietic stem cell transplantation in a child with active disseminated Mycobacterium fortuitum infection and interferon-gamma receptor 1 deficiency. Bone Marrow Transplant. 2006 Jul;38(1):75-6.

[5] Remiszewski P, Roszkowska-Sliz B, Winek J, Chapgier A, Feinberg J, Langfort R, et al. Disseminated Mycobacterium avium infection in a 20-year-old female with partial recessive IFNgammaR1 deficiency. Respiration. 2006;73(3):375-8.

[6] Allende LM, Lopez-Goyanes A, Paz-Artal E, Corell A, Garcia-Perez MA, Varela P, et al. A point mutation in a domain of gamma interferon receptor 1 provokes severe immunodeficiency. Clin Diagn Lab Immunol. 2001 Jan;8(1):133-7.

[7] Okada S, Ishikawa N, Shirao K, Kawaguchi H, Tsumura M, Ohno Y, et al. The novel IFNGR1 mutation 774del4 produces a truncated form of interferon-gamma receptor 1 and has a dominant-negative effect on interferon-gamma signal transduction. J Med Genet. 2007 Aug;44(8):485-91.

[8] Sharma VK, Pai G, Deswarte C, Lodha R, Singh S, Kang LW, et al. Disseminated Mycobacterium avium complex infection in a child with partial dominant interferon gamma receptor 1 deficiency in India. J Clin Immunol. 2015 Jul;35(5):459-62.

[9] Staines-Boone AT, Deswarte C, Venegas Montoya E, Sanchez-Sanchez LM, Garcia Campos JA, Muniz-Ronquillo T, et al. Multifocal Recurrent Osteomyelitis and Hemophagocytic Lymphohistiocytosis in a Boy with Partial Dominant IFN-gammaR1 Deficiency: Case Report and Review of the Literature. Front Pediatr. 2017;5:75.

[10] Rose DM, Atkins J, Holland SM, Infante AJ. A novel mutation in IFN-gamma receptor 1 presenting as multisystem Mycobacterium intracellulare infection. J Allergy Clin Immunol. 2014 Feb;133(2):591-2.

SUPPLEMENTARY TABLE 3.

[1] Jouanguy E, Altare F, Lamhamedi S, Revy P, Emile JF, Newport M, et al. Interferon-gamma-receptor deficiency in an infant with fatal bacille Calmette-Guerin infection. N Engl J Med. 1996 Dec 26;335(26):1956-61.

[2] Kong XF, Vogt G, Chapgier A, Lamaze C, Bustamante J, Prando C, et al. A novel form of cell type-specific partial IFN-gammaR1 deficiency caused by a germ line mutation of the IFNGR1 initiation codon. Hum Mol Genet. 2010 Feb 1;19(3):434-44.

[3] Jouanguy E, Lamhamedi-Cherradi S, Altare F, Fondaneche MC, Tuerlinckx D, Blanche S, et al. Partial interferon-gamma receptor 1 deficiency in a child with tuberculoid bacillus Calmette-Guerin infection and a sibling with clinical tuberculosis. J Clin Invest. 1997 Dec 1;100(11):2658-64.

[4] Jouanguy E, Dupuis S, Pallier A, Doffinger R, Fondaneche MC, Fieschi C, et al. In a novel form of IFN-gamma receptor 1 deficiency, cell surface receptors fail to bind IFN-gamma. J Clin Invest. 2000 May;105(10):1429-36.

[5] Chantrain CF, Bruwier A, Brichard B, Largent V, Chapgier A, Feinberg J, et al. Successful hematopoietic stem cell transplantation in a child with active disseminated Mycobacterium fortuitum infection and interferon-gamma receptor 1 deficiency. Bone Marrow Transplant. 2006 Jul;38(1):75-6.

[6] Okada S, Ishikawa N, Shirao K, Kawaguchi H, Tsumura M, Ohno Y, et al. The novel IFNGR1 mutation 774del4 produces a truncated form of interferon-gamma receptor 1 and has a dominant-negative effect on interferon-gamma signal transduction. J Med Genet. 2007 Aug;44(8):485-91.

[7] Taramasso L, Boisson-Dupuis S, Garre ML, Bondi E, Cama A, Nozza P, et al. Pineal germinoma in a child with interferon-gamma receptor 1 deficiency. case report and literature review. J Clin Immunol. 2014 Nov;34(8):922-7.

[8] Sharma VK, Pai G, Deswarte C, Lodha R, Singh S, Kang LW, et al. Disseminated Mycobacterium avium complex infection in a child with partial dominant interferon gamma receptor 1 deficiency in India. J Clin Immunol. 2015 Jul;35(5):459-62.

[9] Staines-Boone AT, Deswarte C, Venegas Montoya E, Sanchez-Sanchez LM, Garcia Campos JA, Muniz-Ronquillo T, et al. Multifocal Recurrent Osteomyelitis and Hemophagocytic Lymphohistiocytosis in a Boy with Partial Dominant IFN-gammaR1 Deficiency: Case Report and Review of the Literature. Front Pediatr. 2017;5:75.

[10] Rosenzweig S, Dorman SE, Roesler J, Palacios J, Zelazko M, Holland SM. 561del4 defines a novel small deletion hotspot in the interferon-gamma receptor 1 chain. Clin Immunol. 2002 Jan;102(1):25-7.

[11] Allende LM, Lopez-Goyanes A, Paz-Artal E, Corell A, Garcia-Perez MA, Varela P, et al. A point mutation in a domain of gamma interferon receptor 1 provokes severe immunodeficiency. Clin Diagn Lab Immunol. 2001 Jan;8(1):133-7.

[12] Rose DM, Atkins J, Holland SM, Infante AJ. A novel mutation in IFN-gamma receptor 1 presenting as multisystem Mycobacterium intracellulare infection. J Allergy Clin Immunol. 2014 Feb;133(2):591-2.

[13] Villella A, Picard C, Jouanguy E, Dupuis S, Popko S, Abughali N, et al. Recurrent Mycobacterium avium osteomyelitis associated with a novel dominant interferon gamma receptor mutation. Pediatrics. 2001 Apr;107(4):E47.

[14] Galal N, Boutros J, Marsafy A, Kong XF, Feinberg J, Casanova JL, et al. Mendelian susceptibility to mycobacterial disease in egyptian children. Mediterr J Hematol Infect Dis. 2012;4(1):e2012033.

[15] Luo Q, Wang R, Fu Q, Chen F, Tan Z. Novel IFNGR1 Mutation in a Child with Mycobacterium avium Infection. J Clin Immunol. 2023 Nov;43(8):1778-81.

[16] Remiszewski P, Roszkowska-Sliz B, Winek J, Chapgier A, Feinberg J, Langfort R, et al. Disseminated Mycobacterium avium infection in a 20-year-old female with partial recessive IFNgammaR1 deficiency. Respiration. 2006;73(3):375-8.

[17] Sologuren I, Boisson-Dupuis S, Pestano J, Vincent QB, Fernandez-Perez L, Chapgier A, et al. Partial recessive IFN-gammaR1 deficiency: genetic, immunological and clinical features of 14 patients from 11 kindreds. Hum Mol Genet. 2011 Apr 15;20(8):1509-23.

[18] Jouanguy E, Lamhamedi-Cherradi S, Lammas D, Dorman SE, Fondaneche MC, Dupuis S, et al. A human IFNGR1 small deletion hotspot associated with dominant susceptibility to mycobacterial infection. Nat Genet. 1999 Apr;21(4):370-8.

[19] Hoshina T, Takada H, Sasaki-Mihara Y, Kusuhara K, Ohshima K, Okada S, et al. Clinical and host genetic characteristics of Mendelian susceptibility to mycobacterial diseases in Japan. J Clin Immunol. 2011 Jun;31(3):309-14.

[20] Obinata K, Lee T, Niizuma T, Kinoshita K, Shimizu T, Hoshina T, et al. Two cases of partial dominant interferon-gamma receptor 1 deficiency that presented with different clinical courses of bacille Calmette-Guerin multiple osteomyelitis. J Infect Chemother. 2013 Aug;19(4):757-60

SUPPLEMENTARY TABLE 4.

Patients with AR IFNGR1 mutations

[1] Sologuren I, Boisson-Dupuis S, Pestano J, Vincent QB, Fernandez-Perez L, Chapgier A, et al. Partial recessive IFN-gammaR1 deficiency: genetic, immunological and clinical features of 14 patients from 11 kindreds. Hum Mol Genet. 2011 Apr 15;20(8):1509-23.

[2] Chantrain CF, Bruwier A, Brichard B, Largent V, Chapgier A, Feinberg J, et al. Successful hematopoietic stem cell transplantation in a child with active disseminated Mycobacterium fortuitum infection and interferon-gamma receptor 1 deficiency. Bone Marrow Transplant. 2006 Jul;38(1):75-6.

[3] Jouanguy E, Lamhamedi-Cherradi S, Altare F, Fondaneche MC, Tuerlinckx D, Blanche S, et al. Partial interferon-gamma receptor 1 deficiency in a child with tuberculoid bacillus Calmette-Guerin infection and a sibling with clinical tuberculosis. J Clin Invest. 1997 Dec 1;100(11):2658-64.

[4] Jouanguy E, Dupuis S, Pallier A, Doffinger R, Fondaneche MC, Fieschi C, et al. In a novel form of IFN-gamma receptor 1 deficiency, cell surface receptors fail to bind IFN-gamma. J Clin Invest. 2000 May;105(10):1429-36.

[5] Taramasso L, Boisson-Dupuis S, Garre ML, Bondi E, Cama A, Nozza P, et al. Pineal germinoma in a child with interferon-gamma receptor 1 deficiency. case report and literature review. J Clin Immunol. 2014 Nov;34(8):922-7.

[6] Rosenzweig S, Dorman SE, Roesler J, Palacios J, Zelazko M, Holland SM. 561del4 defines a novel small deletion hotspot in the interferon-gamma receptor 1 chain. Clin Immunol. 2002 Jan;102(1):25-7.

[7] Galal N, Boutros J, Marsafy A, Kong XF, Feinberg J, Casanova JL, et al. Mendelian susceptibility to mycobacterial disease in egyptian children. Mediterr J Hematol Infect Dis. 2012;4(1):e2012033.

[8] Remiszewski P, Roszkowska-Sliz B, Winek J, Chapgier A, Feinberg J, Langfort R, et al. Disseminated Mycobacterium avium infection in a 20-year-old female with partial recessive IFNgammaR1 deficiency. Respiration. 2006;73(3):375-8.

Patients with AD IFNGR1 mutations

[9] Okada S, Ishikawa N, Shirao K, Kawaguchi H, Tsumura M, Ohno Y, et al. The novel IFNGR1 mutation 774del4 produces a truncated form of interferon-gamma receptor 1 and has a dominant-negative effect on interferon-gamma signal transduction. J Med Genet. 2007 Aug;44(8):485-91.

[10] Staines-Boone AT, Deswarte C, Venegas Montoya E, Sanchez-Sanchez LM, Garcia Campos JA, Muniz-Ronquillo T, et al. Multifocal Recurrent Osteomyelitis and

Hemophagocytic Lymphohistiocytosis in a Boy with Partial Dominant IFN-gammaR1 Deficiency: Case Report and Review of the Literature. Front Pediatr. 2017;5:75.

[11] Dorman SE, Picard C, Lammas D, Heyne K, van Dissel JT, Baretto R, et al. Clinical features of dominant and recessive interferon gamma receptor 1 deficiencies. Lancet. 2004 Dec 11-17;364(9451):2113-21.

[12] Rose DM, Atkins J, Holland SM, Infante AJ. A novel mutation in IFN-gamma receptor 1 presenting as multisystem Mycobacterium intracellulare infection. J Allergy Clin Immunol. 2014 Feb;133(2):591-2.

[13] Villella A, Picard C, Jouanguy E, Dupuis S, Popko S, Abughali N, et al. Recurrent Mycobacterium avium osteomyelitis associated with a novel dominant interferon gamma receptor mutation. Pediatrics. 2001 Apr;107(4):E47.

[14] Luo Q, Wang R, Fu Q, Chen F, Tan Z. Novel IFNGR1 Mutation in a Child with Mycobacterium avium Infection. J Clin Immunol. 2023 Nov;43(8):1778-81.

[15] Jouanguy E, Lamhamedi-Cherradi S, Lammas D, Dorman SE, Fondaneche MC, Dupuis S, et al. A human IFNGR1 small deletion hotspot associated with dominant susceptibility to mycobacterial infection. Nat Genet. 1999 Apr;21(4):370-8.

[16] Obinata K, Lee T, Niizuma T, Kinoshita K, Shimizu T, Hoshina T, et al. Two cases of partial dominant interferon-gamma receptor 1 deficiency that presented with different clinical courses of bacille Calmette-Guerin multiple osteomyelitis. J Infect Chemother. 2013 Aug;19(4):757-60.

[17] Hoshina T, Takada H, Sasaki-Mihara Y, Kusuhara K, Ohshima K, Okada S, et al. Clinical and host genetic characteristics of Mendelian susceptibility to mycobacterial diseases in Japan. J Clin Immunol. 2011 Jun;31(3):309-14.

Patients with STAT1 GOF mutations

[18] Toubiana J, Okada S, Hiller J, Oleastro M, Lagos Gomez M, Aldave Becerra JC, et al. Heterozygous STAT1 gain-of-function mutations underlie an unexpectedly broad clinical phenotype. Blood. 2016 Jun 23;127(25):3154-64.

[19] Pedraza-Sanchez S, Lezana-Fernandez JL, Gonzalez Y, Martinez-Robles L, Ventura-Ayala ML, Sadowinski-Pine S, et al. Disseminated Tuberculosis and Chronic

Mucocutaneous Candidiasis in a Patient with a Gain-of-Function Mutation in Signal Transduction and Activator of Transcription 1. Front Immunol. 2017;8:1651.

[20] Leiding JW, Okada S, Hagin D, Abinun M, Shcherbina A, Balashov DN, et al. Hematopoietic stem cell transplantation in patients with gain-of-function signal transducer and activator of transcription 1 mutations. J Allergy Clin Immunol. 2018 Feb;141(2):704-17 e5.

Patients with LOF STAT1 mutations

[21] Liu Z, Zhou M, Yuan C, Ni Z, Liu W, Tan Y, et al. Two novel STAT1 mutations cause Mendelian susceptibility to mycobacterial disease. Biochem Biophys Res Commun. 2022 Feb 5;591:124-9.

[22] Hirata O, Okada S, Tsumura M, Kagawa R, Miki M, Kawaguchi H, et al. Heterozygosity for the Y701C STAT1 mutation in a multiplex kindred with multifocal osteomyelitis. Haematologica. 2013 Oct;98(10):1641-9.

[23] Ye F, Zhang W, Dong J, Peng M, Fan C, Deng W, et al. A novel STAT1 loss-of-function mutation associated with Mendelian susceptibility to mycobacterial disease. Front Cell Infect Microbiol. 2022;12:1002140.

[24] Tsumura M, Miki M, Mizoguchi Y, Hirata O, Nishimura S, Tamaura M, et al. Enhanced osteoclastogenesis in patients with MSMD due to impaired response to IFN-gamma. J Allergy Clin Immunol. 2022 Jan;149(1):252-61 e6.

[25] Ueki M, Yamada M, Ito K, Tozawa Y, Morino S, Horikoshi Y, et al. A heterozygous dominant-negative mutation in the coiled-coil domain of STAT1 is the cause of autosomal-dominant Mendelian susceptibility to mycobacterial diseases. Clin Immunol. 2017 Jan;174:24-31.

[26] Kagawa R, Fujiki R, Tsumura M, Sakata S, Nishimura S, Itan Y, et al. Alanine-scanning mutagenesis of human signal transducer and activator of transcription 1 to estimate loss- or gain-of-function variants. J Allergy Clin Immunol. 2017 Jul;140(1):232-41.

[27] Lim QY, Leung D, Lam CK, Yang X, Cheong KN, Yik AKH, et al. Case report: A novel de novo germline loss-of-function mutation in the STAT1 transactivation domain in two Chinese siblings, with the elder sibling presenting with multifocal Bacillus Calmette-Guerin osteomyelitis. Front Immunol. 2024;15:1504816.

Patients with Anti-Interferon Gamma Antibodies (AIGA)

[28] Tang M, Huang J, Zeng W, Huang Y, Lei Y, Qiu Y, et al. Retrospective Analysis of 10 Cases of Disseminated Nontuberculous Mycobacterial Disease with Osteolytic Lesions. Infect Drug Resist. 2021;14:4667-79.

[29] Nishimura T, Fujita-Suzuki Y, Yonemaru M, Ohkusu K, Sakagami T, Carpenter SM, et al. Recurrence of disseminated Mycobacterium avium complex disease in a patient with anti-gamma interferon autoantibodies by reinfection. J Clin Microbiol. 2015 Apr;53(4):1436-8.

[30] Tang M, Zeng W, Qiu Y, Fang G, Pan M, Li W, et al. Clinical features of rare disseminated Mycobacterium colombiense infection in nine patients who are HIV-negative in Guangxi, China. Int J Infect Dis. 2023 Mar;128:321-4.

[31] Zhao L, Ma J, Sun Y, Yu X, Lu Y, Qian H, et al. Deepening Understanding of the Clinical Features and Diagnostic Approaches to Anti-Interferon-Gamma Autoantibody Associated Adult-Onset Immunodeficiency in the Last 20 Years: A Case Report and Literature Review. J Clin Immunol. 2025 May 13;45(1):93.
