# Supplementary material for: Mycobacterial infections and recurrent osteomyelitis in a patient with an autosomal dominant mutation in IFN-gamma receptor 1 with overexpression of the receptor
Source: Int J Infect Dis. Author manuscript; Available in PMC 2026 Nov 5. (PMC13739071; doi:10.1016/j.ijid.2026.109104)
Supplement: Supplementary file 2 [file NIHMS2218719-supplement-Supplementary_Figure_S1.pdf]

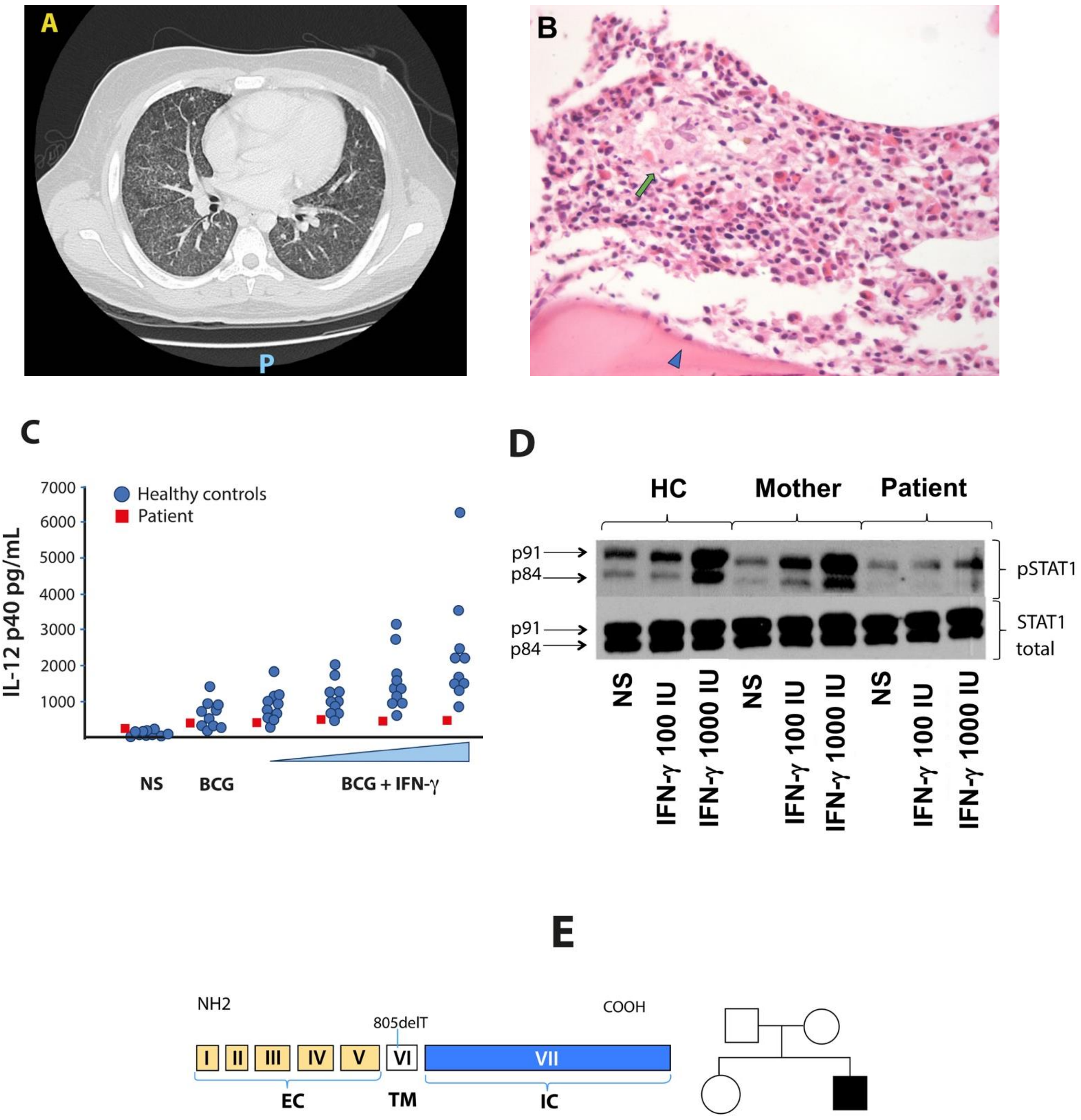


**Supplementary Figure S1. A** Thorax CT showing nodular lesions in both lungs. **B** Osteoblast rimming a bone spicule (arrowhead) with a paratrabecular cluster of epithelioid histiocytes (arrow) admixed with myeloid precursors **C** IL-12 production by diluted whole blood, from healthy donors (n = 10, blue points) and patient (red square), stimulated as indicated and assessed by ELISA in 48h supernatants. NS, Non-stimulated **D** p-Stat-1 detected by Western Blot in whole cell lysates from EBV-LCL from patient, his mother and a healthy control, stimulated by 20 minutes as indicated **E** Schematic representation of the IFN-γR1 structure and the position of the mutation. EC, TM and IC, extracellular, transmembrane and intracellular domains, respectively. The family pedigree shows that the patient was the only carrier of pathogenic variant in *IFNGR1*.
