# Supplementary material for: Mycobacterial infections and recurrent osteomyelitis in a patient with an autosomal dominant mutation in IFN-gamma receptor 1 with overexpression of the receptor
Source: Int J Infect Dis. Author manuscript; Available in PMC 2026 Nov 5. (PMC13739071; doi:10.1016/j.ijid.2026.109104)
Supplement: Supplementary file 3 [file NIHMS2218719-supplement-Supplementary_Figure_S2.pdf]

Flow cytometry results supporting evidence of mutation on *IFNGR1* in the studied patient.

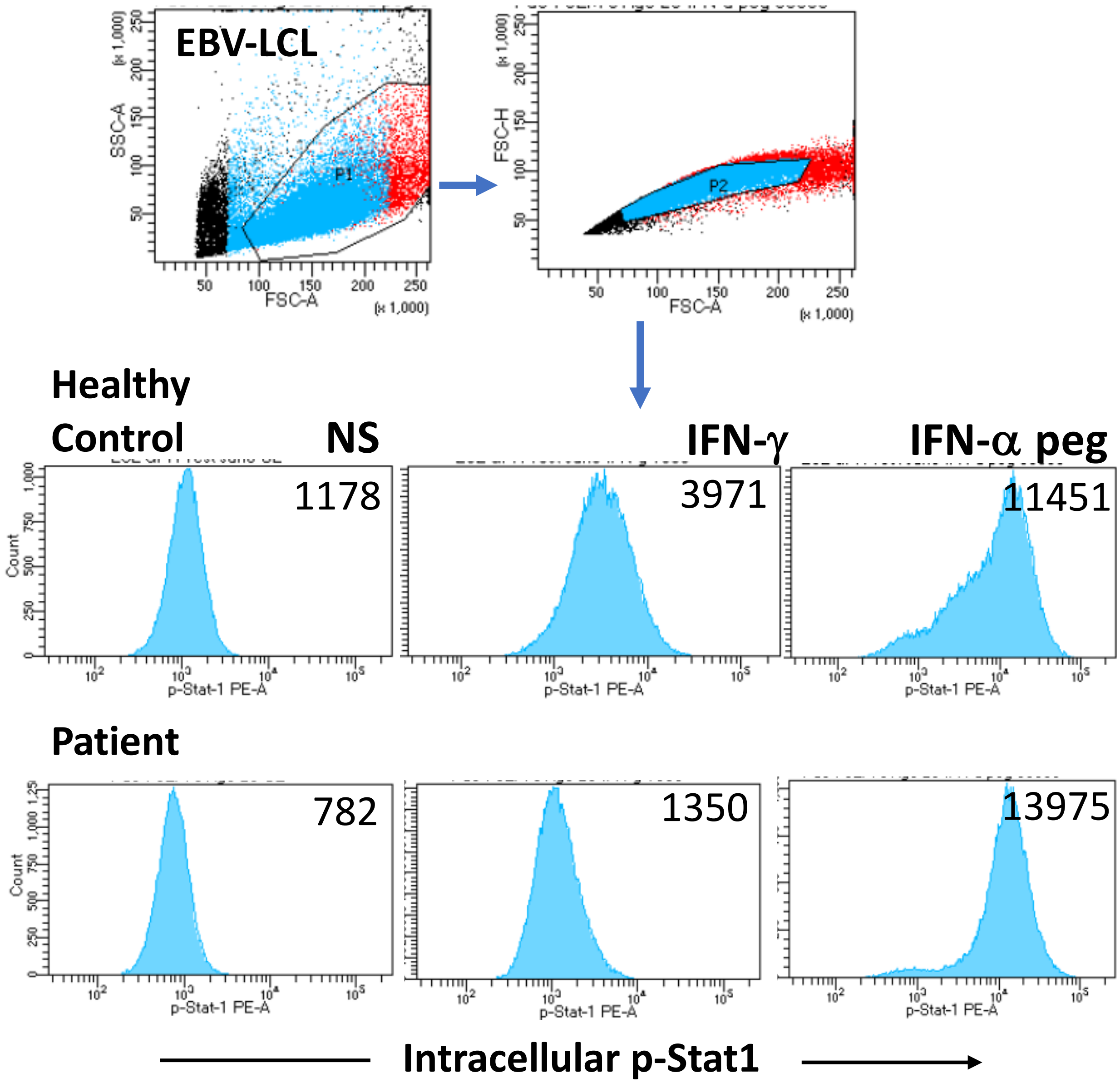

Supplementary figure S2. Phosphorylation of Stat-1 on Epstein-Barr Virus Lymphoblastoid Cell Lines (EBV-LCL) from patient and a Healthy control, responding to IFN-γ and IFN-α, assessed by flow cytometry. LCL were stimulated with 1000 IU of IFN-g or 25000 IU of Pegylated IFN-α for 20 minutes. Then p-Stat-1 was detected by intracellular staining using an anti-pStat-1 PE antibody, and analyzed in a FACSCanto flow cytometer. The upper dot plot graphics represent plots of size vs granularity of the LCL and the lower histograms correspond to the expression of IFN-γ R1 in the cells from control and the patient. Stimulated as indicated. The numbers in histograms correspond to fluorescence values for intracellular p-Stat-1. NS= Non stimulated
