# Supplementary material for: Mycobacterial infections and recurrent osteomyelitis in a patient with an autosomal dominant mutation in IFN-gamma receptor 1 with overexpression of the receptor
Source: Int J Infect Dis. Author manuscript; Available in PMC 2026 Nov 5. (PMC13739071; doi:10.1016/j.ijid.2026.109104)
Supplement: Supplementary file 4 [file NIHMS2218719-supplement-Supplementary_Figure_S3.pdf]

A)

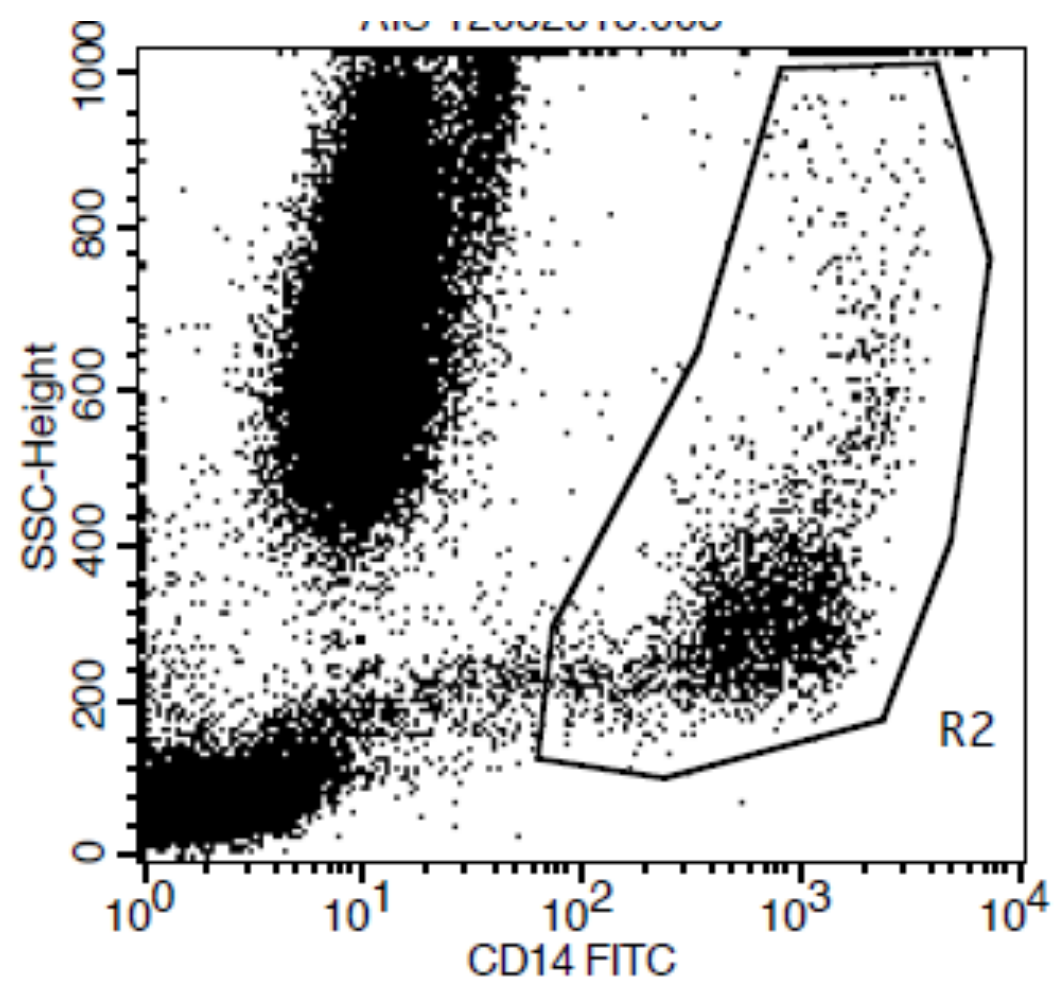

B)

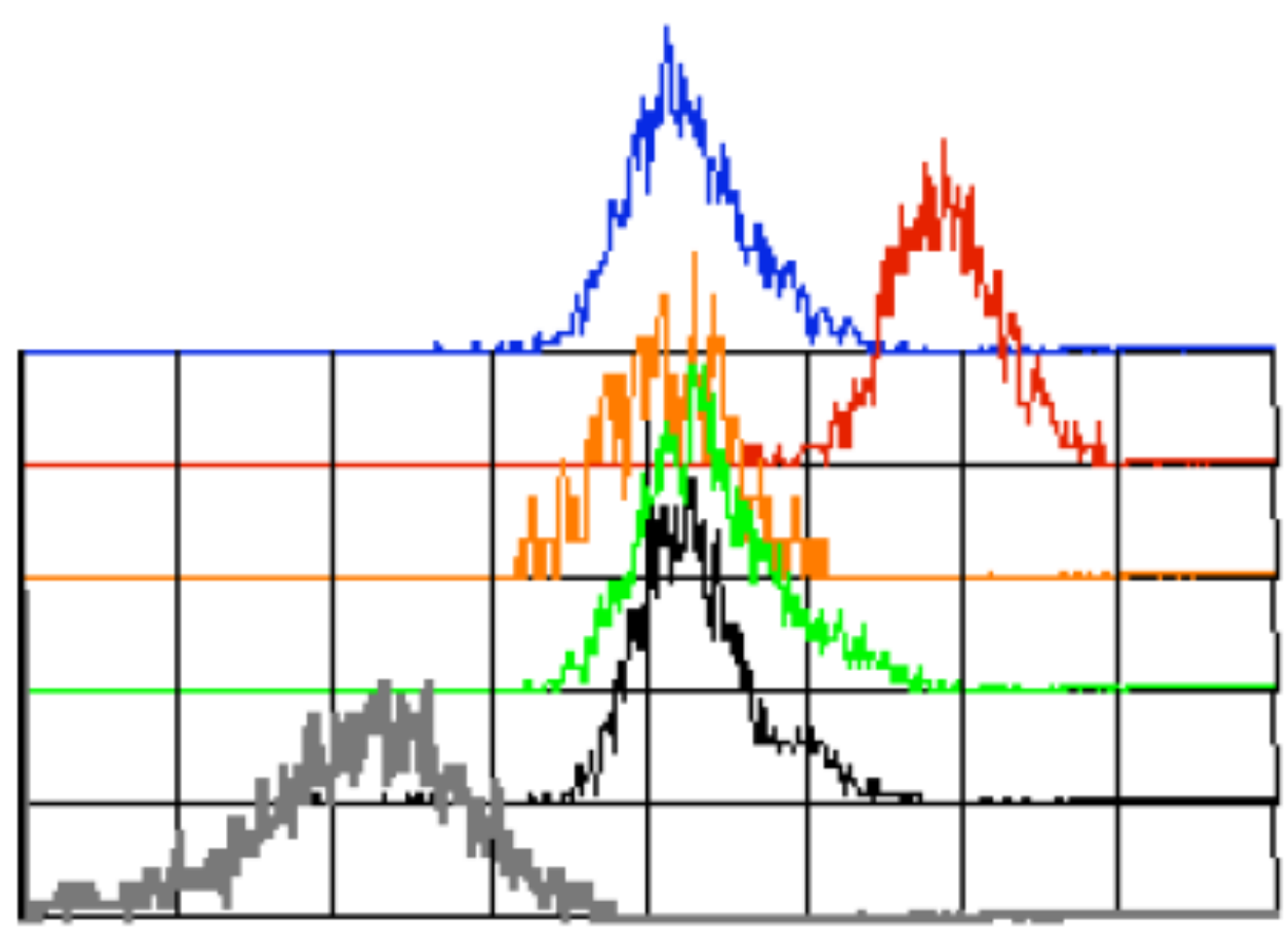

Supplementary figure S3. Expression of CD119 or IFN-γ R1 on CD14+ blood monocytes from patient, relatives and control. Whole blood from patient and distinct donors were labelled on membrane with CD14 and CD119 antibodies, and erythrocytes were lysed for analysis. A) A gate in CD14+ monocytes were selected in a dot plot as shown, and analysed for IFN-γ R1 fluorescence in histograms of panel B). B) histograms for CD119 expression: grey, isotype control cells from healthy donor, black cells from patient´s father; green cells from patient´s sister, orange and blue, cells from unrelated controls. Red, cells from patient with *IFNGR1* mutation.
