# Supplementary material for: Mycobacterial infections and recurrent osteomyelitis in a patient with an autosomal dominant mutation in IFN-gamma receptor 1 with overexpression of the receptor
Source: Int J Infect Dis. Author manuscript; Available in PMC 2026 Nov 5. (PMC13739071; doi:10.1016/j.ijid.2026.109104)
Supplement: Supplementary file 5 [file NIHMS2218719-supplement-Supplementary_Figure_S4.pdf]

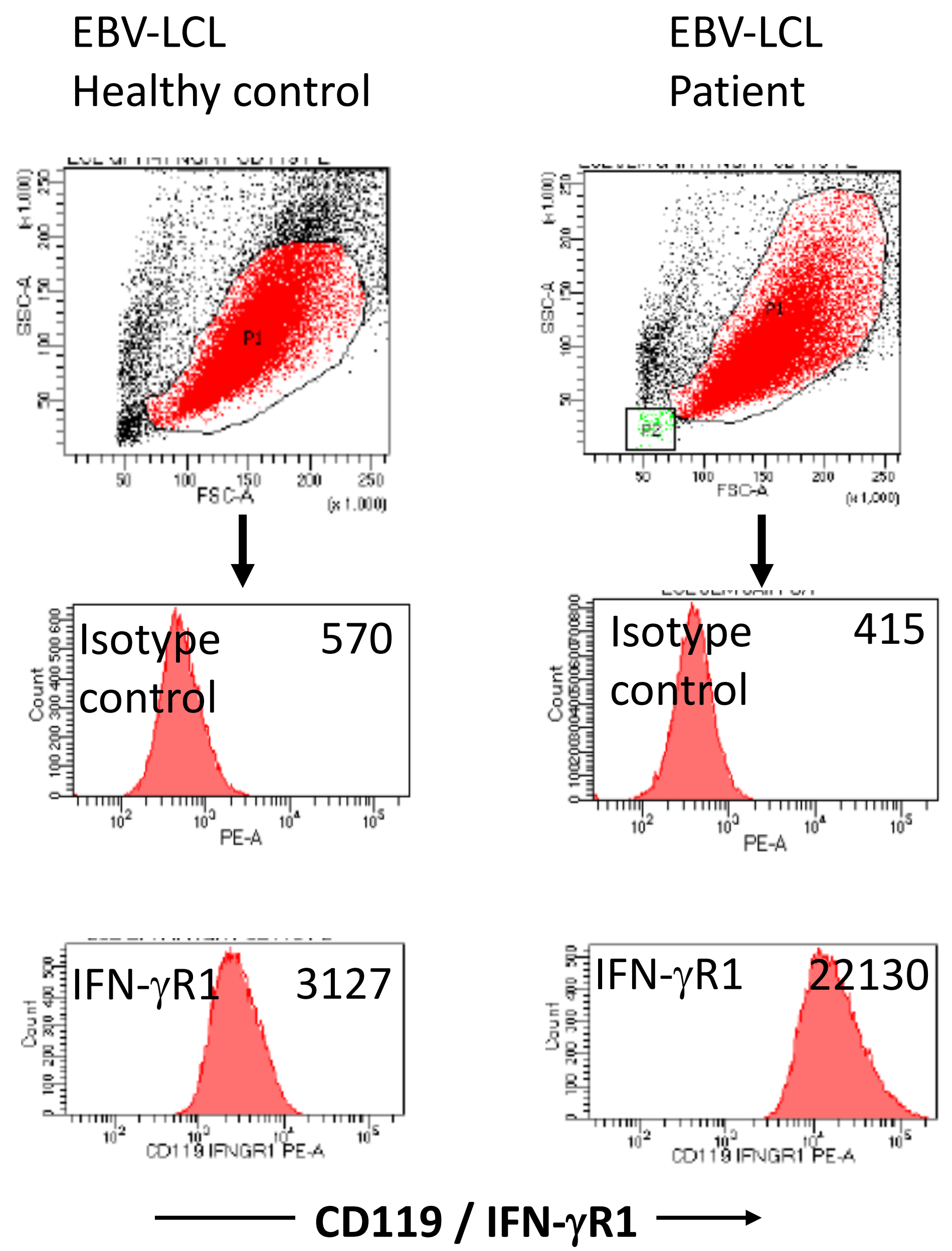

Supplementary figure S4. **Expression of CD119 / IFN-γ R1 on Epstein-Barr Virus Lymphoblastoid Cell Lines (EBV-LCL) from patient and a Healthy control.** LCL were established after infection of blood mononuclear cells from donors, with EBV, and expression of CD119 / IFN-gR1 was assessed by flow cytometry. The upper dot plot graphics represent plots of size vs granularity of the LCL, and the lower histograms correspond to the expression of IFN-γ R1 in the cells from control and the patient. The numbers in histograms are the fluorescence values for the isotype controls and IFN-γ R. CD119 is expressed 7fold in patient´s cells
