# Supplementary material for: Mycobacterial infections and recurrent osteomyelitis in a patient with an autosomal dominant mutation in IFN-gamma receptor 1 with overexpression of the receptor
Source: Int J Infect Dis. Author manuscript; Available in PMC 2026 Nov 5. (PMC13739071; doi:10.1016/j.ijid.2026.109104)
Supplement: Supplementary file 6 [file NIHMS2218719-supplement-Supplementary_Figure_S5.pdf]

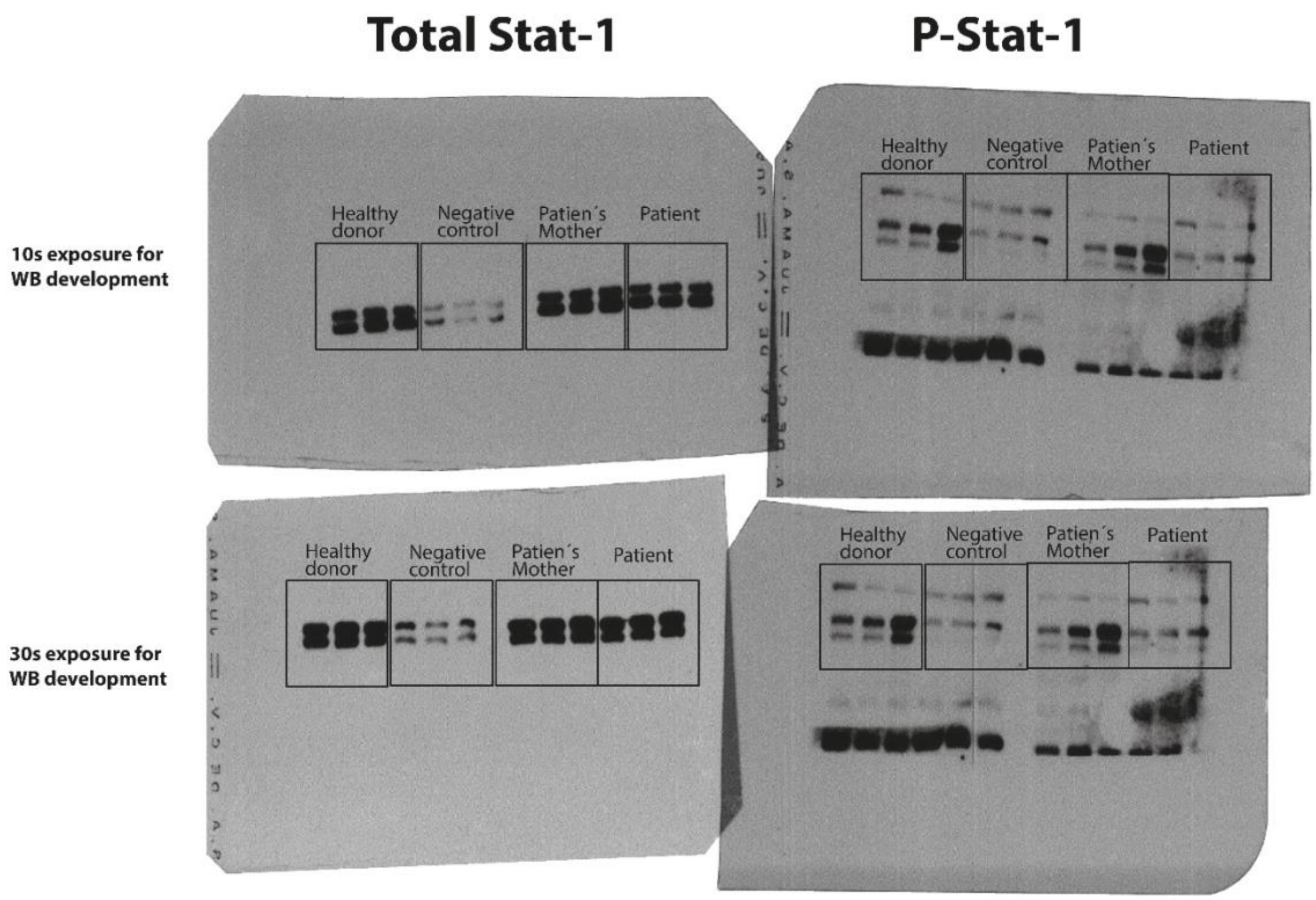

Supplementary Figure S5. Non-edited Western Blot for Stat-1 and p-Stat-1 from whole cell lysates from patients and controls. EBV-LCL from a healthy positive control, negative control (from a second patient with a distinct *IFNGR1* heterozygous variant), the patient´s mother and the patient, were incubated during 20 minutes with no stimulus or 100 or 1000 IU IFN-gamma, and reaction was stopped with paraformaldehyde. Whole cell lysates were obtained and Total Stat-1 and phospho -Stat-1 (p-Stat-1) were detected by western blot, as previously published (Pedraza-Sanchez, Lezana JL, Gonzalez Y et al, 2017. Front Immunol. 8:1651). The order of the lines are the same for all the donors, from left to right: unstimulated, 100 IU IFN-gamma and 1000 IU IFN-gamma. The figure shows the same film developed with distinct exposure times. The experiment was repeated three times.
