# Supplementary material for: Mycobacterial infections and recurrent osteomyelitis in a patient with an autosomal dominant mutation in IFN-gamma receptor 1 with overexpression of the receptor
Source: Int J Infect Dis. Author manuscript; Available in PMC 2026 Nov 5. (PMC13739071; doi:10.1016/j.ijid.2026.109104)
Supplement: Supplementary file 7 [file NIHMS2218719-supplement-Supplementary_Figure_S6.pdf]

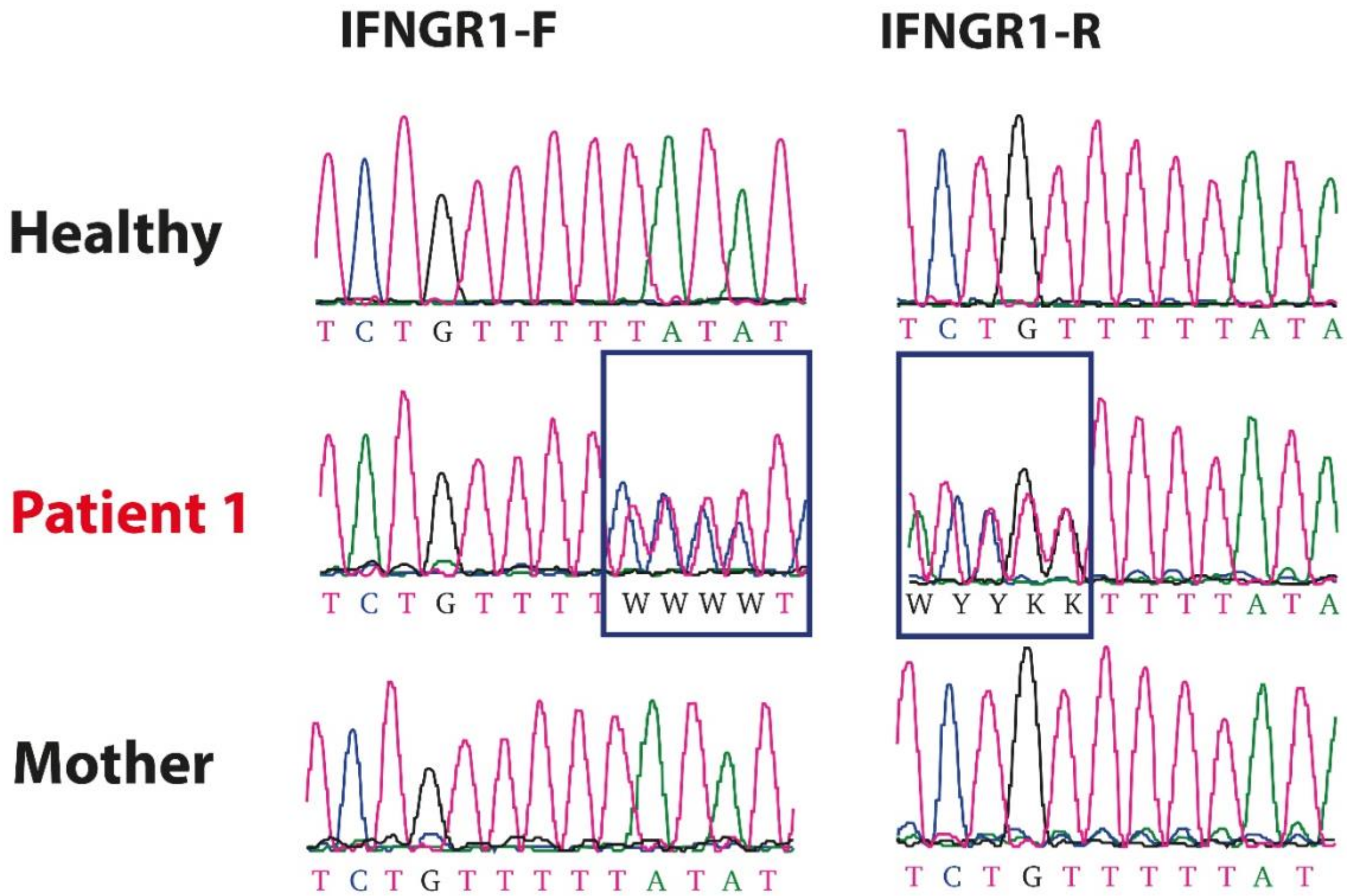

Supplementary Figure S6. Sanger sequencing of the *IFNGR1* c.805delT (p.Tyr269Ilefs*8) variant. Genomic DNA from EBV-transformed lymphoblastoid cell lines (EBV-LCLs) was extracted using the phenol-chloroform method. The seven exons of *IFNGR1* and the 22 exons of *STAT1* were amplified by PCR using specific forward and reverse primers. PCR products were recovered from agarose gels and sequenced using the BigDye Terminator kit on an ABI Prism 377 Sequence Analyzer (PerkinElmer Systems). Sequences were compared with the corresponding reference sequences using SeqMan Pro (DNASTAR). Representative electropherograms show the heterozygous *IFNGR1* c.805delT (p.Tyr269Ilefs*8) variant in the proband and its absence in both parents, supporting a *de novo* occurrence.
