# Supplementary material for: Mycobacterial infections and recurrent osteomyelitis in a patient with an autosomal dominant mutation in IFN-gamma receptor 1 with overexpression of the receptor
Source: Int J Infect Dis. Author manuscript; Available in PMC 2026 Nov 5. (PMC13739071; doi:10.1016/j.ijid.2026.109104)
Supplement: Supplementary file 8 [file NIHMS2218719-supplement-Supplementary_Figure_S7.pdf]

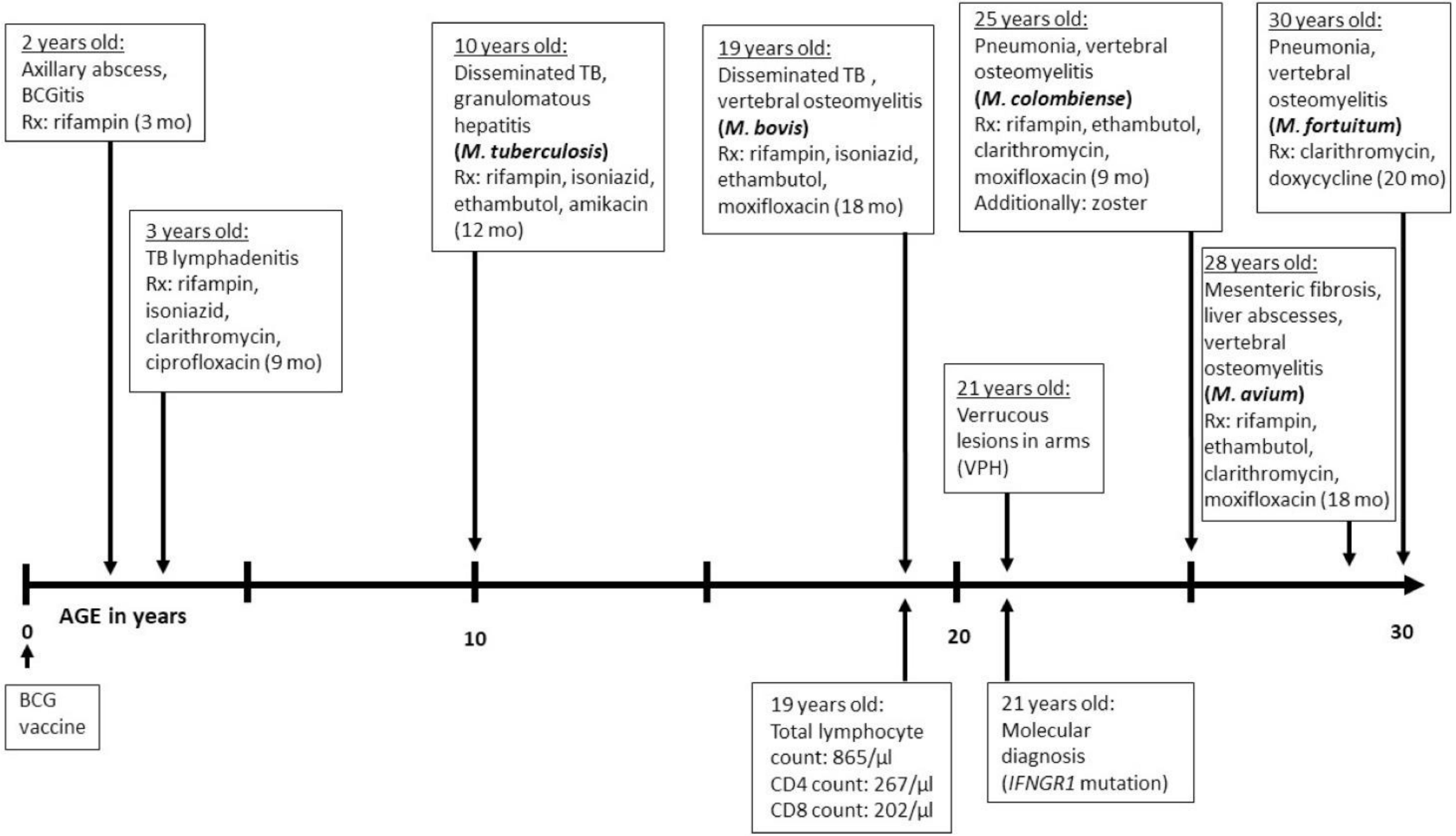
2 years old:
Axillary abscess, BCGitis
Rx: rifampin (3 mo)
3 years old:
TB lymphadenitis
Rx: rifampin, isoniazid, clarithromycin, ciprofloxacin (9 mo)
10 years old:
Disseminated TB, granulomatous hepatitis
(M. tuberculosis)
Rx: rifampin, isoniazid, ethambutol, amikacin (12 mo)
19 years old:
Disseminated TB , vertebral osteomyelitis
(M. bovis)
Rx: rifampin, isoniazid, ethambutol, moxifloxacin (18 mo)
25 years old:
Pneumonia, vertebral osteomyelitis
(M. colombiense)
Rx: rifampin, ethambutol, clarithromycin, moxifloxacin (9 mo)
Additionally: zoster
30 years old:
Pneumonia, vertebral osteomyelitis
(M. fortuitum)
Rx: clarithromycin, doxycycline (20 mo)
28 years old:
Mesenteric fibrosis, liver abscesses, vertebral osteomyelitis
(M. avium)
Rx: rifampin, ethambutol, clarithromycin, moxifloxacin (18 mo)
21 years old:
Verrucous lesions in arms (VPH)
AGE in years
0
10
20
30
BCG vaccine
19 years old:
Total lymphocyte count: 865/µl
CD4 count: 267/µl
CD8 count: 202/µl
21 years old:
Molecular diagnosis
(IFNGR1 mutation)

Supplementary Figure S7. Timeline of the patient presented in the clinical case. The main clinical symptoms, proved infections and treatments are shown.
