# Supplementary material for: Mycobacterial infections and recurrent osteomyelitis in a patient with an autosomal dominant mutation in IFN-gamma receptor 1 with overexpression of the receptor
Source: Int J Infect Dis. Author manuscript; Available in PMC 2026 Nov 5. (PMC13739071; doi:10.1016/j.ijid.2026.109104)
Supplement: Supplementary file 9 [file NIHMS2218719-supplement-Supplementary_Figure_S8.pdf]

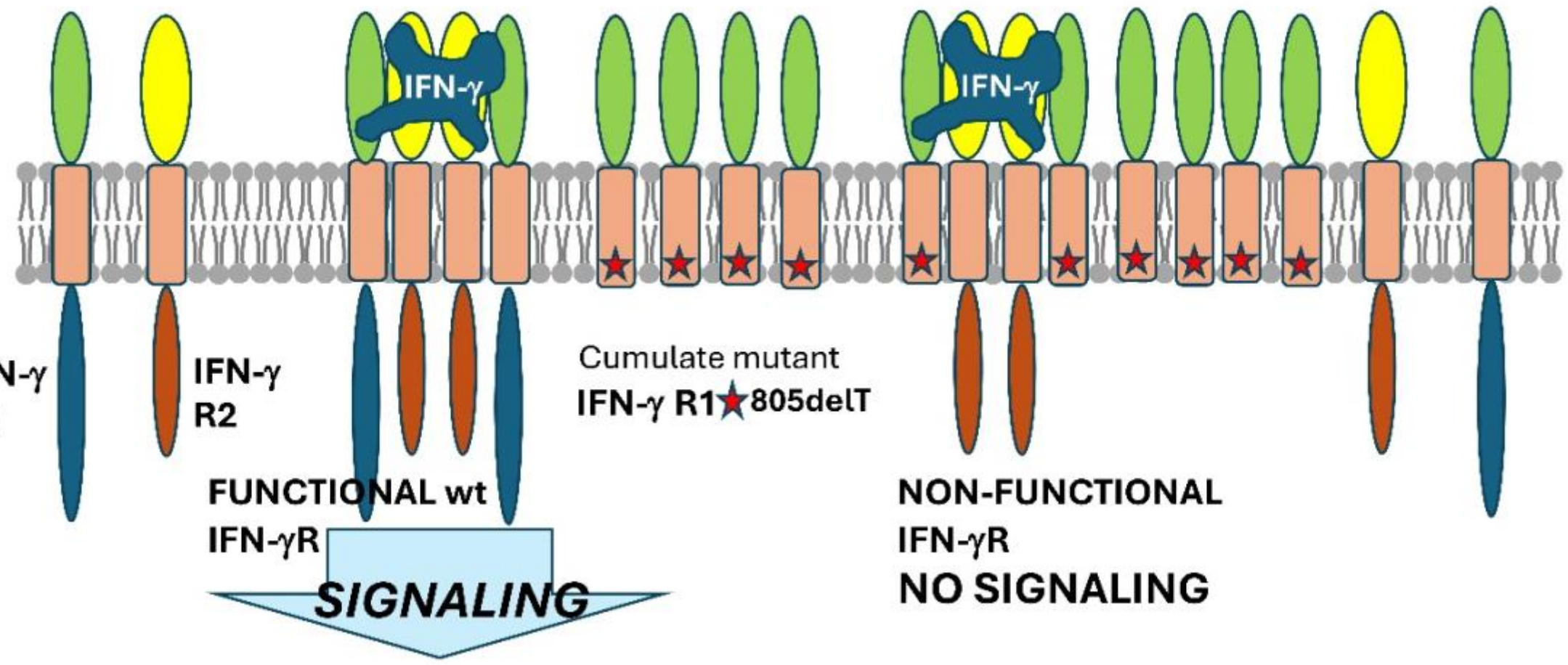

Supplementary Figure S8. Model of mutant IFN-γ Receptor 1 and mechanism of dominant negative effect. In the left part of the figure, WT IFN-γR1 and WT IFN-γR2 form a tetrameric receptor that binds IFN-γ and induces normal downstream signaling required for phagocyte activation. At the right, the c.805-delT mutation prevents complete transcription of the polypeptide and disrupts the recycling motif of IFN-γR1 (red star), resulting in accumulation of a truncated receptor at the cell membrane. These mutant receptors can still dimerize with WT IFN-γR2 and can bind IFN-γ, but are unable to signal, thereby impairing IFN-γ mediated cellular responses.
