# Supplementary material for: Mycobacterial infections and recurrent osteomyelitis in a patient with an autosomal dominant mutation in IFN-gamma receptor 1 with overexpression of the receptor
Source: Int J Infect Dis. Author manuscript; Available in PMC 2026 Nov 5. (PMC13739071; doi:10.1016/j.ijid.2026.109104)
Supplement: Supplementary file 10 [file NIHMS2218719-supplement-Supplementary_Methodology.pdf]

Mycobacterial infections and recurrent osteomyelitis in a patient with an autosomal dominant mutation in IFN-gamma receptor 1 with overexpression of the receptor.

Pamela Rivero-García[1], Luis Martínez-Robles[2, 6], Yolanda González[3], Laura Ventura-Ayala[2], Eric Ochoa-Hein[4], Armando Gamboa-Domínguez[5], Jean-Laurent Casanova[7-11], Stephanie Boisson-Dupuis[7-9], Sigifredo Pedraza-Sánchez[2, #].

Supplementary methodology for flow cytometry and Western Blot.

Membrane assessment of CD119 (IFN-γ R1) in cells from patient and controls, by flow cytometry. Peripheral Mononuclear Cells (PBMC) were obtained from blood of patient and control donors. Cells were labelled with anti CD14 FITC antibody (Becton Dickinson BD 555397) and with anti CD119 (IFN-gR1)- PE (BD Cat 558937) during 20 minutes at room temperature in darkness, following of lysis of erythrocytes. Cells were washed by centrifugation 5 min at 300 x g and resuspended in 1% paraformaldehyde in PBS. Cells were acquired and analysed in a BD FACSort flow cytometer. In addition, CD119 /IFN-γR was also assessed on EBV-LCL (Epstein-Barr Virus transformed Lymphoblastoid Cell Lines) from patient and healthy controls, using the same anti-CD119 PE antibody to label the receptor on blood monocytes, and acquired and analysed in a BD FACSCanto flow cytometer. Analysis were done using Cell Quest or FACSDiva software. Assessment of receptor was repeated two times for blood leukocytes and three times in EBV-LCL.

Intracellular assessment of p-Stat-1 in EBV-LCL. To assess intracellularly p-Stat-1 in cells from patient and controls, responding to IFN-γ, we follow the methodology described in the literature a) Dorman and Holland, 1998, J Clin Invest, 101:2364-69; b) Ilanguman S, et al, 2003. J Immunol Methods, 278:221-. Briefly, EBV-LCL were first cultured to 70% confluency in RPMI+10% FBS. The day before the experiment, cells were cultured in RPMI+2% FBS and the day of the experiment, cells were starved in Serum-free RPMI for two hours. Cells were placed in FACS tubes at 0.5 million cells per condition, and stimulated for 20 minutes with 1000 IU of recombinant human IFN-γ (R&D cat 285 IF), with no stimulus and with rh IFN-alpha (pegylated IFN-α, for human use Pegasys (Roche U.S.A.)). Phosphorylation was stopped adding 0.5 mL of 4% paraformaldehyde in PBS, incubating 5 additional minutes at 37°C in water bath, and cells were washed by centrifugation. Supernatants were discarded, and cells were permeabilized

during 15 minutes with 1 mL of permeabilizing buffer (FACS Perm2 buffer BD Cat.349692) Prepared diluting the 10X stock, in methanol); cells were washed twice with PBA (PBS + 1% FBS and 0.1% NaN3) and resuspended in 1% - Paraformaldehyde in PBS, and analysed in a FACS canto flow cytometer. At least 30,000 events were recorded. Analysis were done using FACS Diva software (Becton-Dickinson),

Detection of Stat-1 and Phospho-Stat-1 by Western Blotting.

The methodology we followed for Western Blotting, is described in : *Pedraza-Sanchez S et al. 2017. Front Immunol 8:1651*. EBV-LCL cells were cultured in RPMI 1640 supplemented with 10% FBS at 37°C and 5% CO_2. One day before the experiment, EBV-LCL were starved in RPMI with 1% of FBS; the next day cells were washed and placed ($5x10^6$ cells for each condition) in 2 mL tubes in serum-free RPMI media, and incubated for two hours at 37ºC and 5% CO_2. After that, cells were stimulated with 100 and 1,000 IU IFN-γ, or 25,000 IU IFN-α (R&D or pegylated IFN-α Pegasys Roche) for 30 min at 37°C. For protein-kinase inhibitor assay, $5x10^6$ EBV-LCLs in one ml of RPMI with 1% FBS were stimulated with 1,000 IU IFN-γ for 30 min and 1µM of staurosporine were added for 15, 30 and 60 min. After, 1 mL of cold PBS was added, cells were centrifuged at 17,000 x g for 1 min at 4°C and lysis of cytoplasmic and nuclear membranes were performed using a membrane lysis buffer (10 mM HEPES [pH 7.9], 10 mM KCl, 0.1 mM EDTA, 0.1 mM EGTA, 0.05% NP-40, 25 mM NaF, 1 mM Na_3VO_4, 1 mM PMSF) and nuclear lysis buffer (20 mM HEPES [pH 7.9], 0.4 M NaCl, 1 mM EDTA, 1 mM EGTA, 25% glycerol, 1 mM PMSF, 25 mM NaF, 1 mM Na_3VO_4). Proteins from whole cell or nuclear lysates were subjected to SDS-PAGE and transferred to a polyvinylidene difluoride membrane (Millipore, Merck, Billerica, MA, U.S.A.). Immunoblotting were performed with anti-phospho-STAT1 Y701 (Cell Signalling), anti-STAT1 and anti-lamin B ot anti-b actin (Santa Cruz, Dallas, TX, U.S.A.) and detected with horseradish peroxidase-conjugated (HRP) goat anti-rabbit IgG (Thermo Scientific). SuperSignal (Thermo Fisher Scientific, Waltham MA, U.S.A.) was used as substrate for HRP.
