# Supplementary material for: Mycobacterial infections and recurrent osteomyelitis in a patient with an autosomal dominant mutation in IFN-gamma receptor 1 with overexpression of the receptor
Source: Int J Infect Dis. Author manuscript; Available in PMC 2026 Nov 5. (PMC13739071; doi:10.1016/j.ijid.2026.109104)
Supplement: Supplementary file 11 [file NIHMS2218719-supplement-Supplementary_Table_S1.pdf]

Supplementary Table 1. Genetic etiologies in humans, related to mutations in the *IFNGR1* gene

Gene	Inheritance	Defect	Protein	Nucleotide	Protein	Published as	Exon	Reference
IFNGR1	AR	Complete	+	c.295_306del	p.Trp99_Val102del	295del12	III	Jouanguy E, 2000
				c.563_566del	p.Leu188Argfs*14	561del4	V	Rosenzweig S, 2002
	AR	Complete	-	c.131del	p.Pro44Leufs*18	131delC	II	Jouanguy E, 1996
				c.230G>A	p.Cys77Tyr	C77F	III	Chantrain CF, 2006
	AR	Partial	+	c.260T>C	p.Ile87Thr	I87T	III	Remiszewski P, 2006
				c.188T>G	p.Val63Gly	V63G	II	Allende LM, 2001
	AD	Partial	+++	c.774_777del	p.Phe258Leufs*18	774del4	VI	Okada S, 2007
				c.819del	p.Asn274Ilefs*3	818delT	VI	Sharma VK, 2015
				c.819_822del	p.Asn274Hisfs*2	818del4	VI	Staines-Boone AT, 2017
				c.805del	p.Tyr269Ilefs*8	805delT	VI	Rose DM, 2014

AD = Autosomic Dominant.   AR = Autosomic Recesive.
