# Supplementary material for: Mycobacterial infections and recurrent osteomyelitis in a patient with an autosomal dominant mutation in IFN-gamma receptor 1 with overexpression of the receptor
Source: Int J Infect Dis. Author manuscript; Available in PMC 2026 Nov 5. (PMC13739071; doi:10.1016/j.ijid.2026.109104)
Supplement: Supplementary file 12 [file NIHMS2218719-supplement-Supplementary_Table_S2.pdf]

Supplementary Table S2 Comparison of clinical and functional characteristics of patients carrying the *IFNGR1* c.805delT (p.Tyr269Ilefs*8) mutation.

Feature	Rose et al., 2014	Current report
Variant in *IFNGR1*	c.805delT (p-Tyr269Ilefs*8) / wt (heterozygous)	c.805delT (p-Tyr269Ilefs*8) / wt (heterozygous)
Inheritance	*De novo* mutation, which. behaves as Autosomic Dominant.	*De novo* mutation, which. behaves as Autosomic Dominant.
Age of onset and clinical follow-up	15 months / 22 months	2 years / 30 years old
First manifestation	Pneumonia and disseminated *M. intracellulare*	BCGitis after vaccination
Mycobacterial infections	*M. intracellulare*	*M. bovis, M. colombiense, M. avium, M. fortuitum*
Osteomyelitis	Multifocal (ribs, long bones, skull)	Multifocal vertebral osteomyelitis (Pott disease) with recurrent bone involvement
Other infections	None reported	Candidemia, *Pseudomonas* sepsis, *E. coli* pyelonephritis. HPV (Human Papilloma Virus) in arms
Functional findings	Increased IFN-γR1 expression	Increased IFN-γR1 expression, impaired Stat-1 phosphorylation, defective IL-12p40 production
Treatment	Multidrug antimycobacterial therapy + prophylaxis	Repeated prolonged antimycobacterial scheme, antifungal therapy, surgery, long-term follow-up
Outcome	Good clinical evolution	Alive in adulthood with recurrent infections controlled under antimicrobial therapy
Overall contribution	First description of the c.805delT variant causing autosomal dominant partial IFN-γR1 deficiency	Expands the phenotypic spectrum, documents more than two decades of disease evolution, includes the associated mycobacterial infections, and provides detailed functional evidence supporting the pathogenic mechanism.
