# Supplementary material for: Mycobacterial infections and recurrent osteomyelitis in a patient with an autosomal dominant mutation in IFN-gamma receptor 1 with overexpression of the receptor
Source: Int J Infect Dis. Author manuscript; Available in PMC 2026 Nov 5. (PMC13739071; doi:10.1016/j.ijid.2026.109104)
Supplement: Supplementary file 13 [file NIHMS2218719-supplement-Supplementary_Table_S3.pdf]

Supplementary Table S3. Clinical and molecular characteristics of patients published in literature with inborn errors of immunity related to mutations in the *IFNGR1* gene

Patient number	Sex	Age of onset	Ethnicity	BCG vaccine	Nucleotide	Protein	Published also as	Consequence	Zygosity	Exon	Defect Inheritance	Protein expression	Infections	Clinical classification	Lab findings	Reference
**1**	F	2.5 mo	Tunisian	Vaccinated	c.131del	p.Pro44Leufs*18	P44Lfs*18	Frameshift	Homozygous	II	AR-complete	-	Regional adenitis, cachexia granuloma atous dermatitis, hepatosplenomegaly, lymph-node enlargement, diffuse pneumonitis, multiple osteolytic lesions. *M. bovis* (BCG strain) identified in lung and bone marrow cultures		Lymph node and skin biopsies revealed ill-circumscribed aggregates of macrophages filled with acid-fast bacilli	Jouanguy E, 1996
**2**	M	3 mo	Finnish	Vaccinated	c.2T>A	p.Met1Lys	M1K	Missense	Homozygous	I	AR-partial	+	Disseminated BCG infection, *M. avium* infection, osteomyelitis of the femur and pelvis	Disseminated BCG infection and disseminated *M. avium* and *M. intracellulare* infection	Very low IFNGR1 expression, no response to IFN-γ with low IL-12 production	Kong FI. 2010
**3**	M	10 mo	Portuguese	Vaccinated	c.260T>C	p.Ile87Thr	I87T	Missense	Homozygous	III	AR-partial	+	BCG infection, tuberculoid skin and lymph granulomas, disseminated *Salmonella enteritidis*, pneumonitis typical of *M. pneumoniae*	Disseminated BCG infection	Normal IFNGR1 expression, partial IFNGR1 response to high IFN-γ concentrations	Jouanguy E, 1997
**4**	F	3 y	Portuguese	No	c.260T>C	p.Ile87Thr	I87T	Missense	Homozygous	III	AR-partial	+	Erythema nodosa, symptomatic primary tuberculosis, pneumonitis typical of *M. pneumoniae*	Disseminated BCG infection	Normal IFNGR1 expression, partial IFNGR1 response to high IFN-γ concentrations	Jouanguy E, 1997
**5**	F	1 y	Algerian	Vaccinated	c.295_306del	p.Trp99_Val102del	295del12, W99_V102del	Inframe deletion	Homozygous	III	AR-complete	+	Disseminated BCG infection, *M. avium* infection	Disseminated BCG infection	Normal IFNGR1 expression, no IFN-γ binding, null IFNGR1 response to high IFN-γ concentrations	Jouanguy E, 2000
**6**	F	NA	Turkish	Vaccinated	c.230G>A	p.Cys77Tyr	C77Y	Missense	Homozygous	III	AR-complete	+	Disseminated BCG infection, *M. fortuitum* infection	Disseminated BCG infection	Normal IFNGR1 expression, null IFNGR1 response to high IFN-γ concentrations	Jouanguy E, 2000
**7**	M	NA	Turkish	Vaccinated	c.230G>A	p.Cys77Tyr	C77Y	Missense	Homozygous	III	AR-complete	+	Disseminated BCG infection, *M. fortuitum* infection	Disseminated BCG infection	Normal IFNGR1 expression, null IFNGR1 response to high IFN-γ concentrations	Jouanguy E, 2000

**8**	F	NA	French/ Portuguese	Vaccinated	c.653_655del, c.181_182delinsCA	p.Glu218del, p.Val61Gln	652del3, V61Q	Inframe deletion, Missense	Compund Heterozygous	I and II	AR-complete	+	Disseminated BCG infection	Disseminated BCG infection	Normal IFNGR1 expression, null IFNGR1 response to high IFN-γ concentrations	Jouanguy E, 2000
**9**	M	2 y	NA	NA	c.230G>A	p.Cys77Tyr	C77Y	Missense	Homozygous	III	AR-complete	-	Disseminated lymphadenopathies, hepatosplenomegaly, mediastinal lymph nodes, *M. fortuitum* isolated from lymph node tissue culture	*M. fortuitum* lymphatic infection	Two positive Mantoux tuberculin skin tests, increased plasma IFN-γ levels	Chantrain CF, 2006
**10**	F	6 mo	Japanese	Vaccinated	c.774_777del	p.Phe258Leufs*18	774del4	Frameshift	Heterozygous	VI	AD-partial	+++	BCG lymphadenitis, multifocal osteomyelitis due to *M. avium*	*M. avium* osteomyelitis	CD14+ cells produced lower levels of TNF-α in response to IFN-γ stimulation, reduced pSTAT1	Okada S, 2007
**11**	M	2 y	Italian	NA	c.523del	p.Tyr175Metfs*2	523delT, Y175Mfs*2	Frameshift	Homozygous	IV	AR-complete	NA	Multi-focal osteomyelitis by *M. scrofulaceum*, enlarged mesenteric and peripancreatic lymph nodes with *M. scrofulaceum* isolated, recurrence of disseminated infection, pineal germinoma	Pineal germinoma, mycobacterial osteomyelitis	Plasma IFN-γ level: 780 pg/mL	Taramasso L, 2014
**12**	M	1 mo	Indian	Vaccinated	c.819del	p.Asn274Ilefs*3	818delT, N274Ifs*3	Frameshift	Heterozygous	VI	AD-partial	NA	Axillary granulomatous lymphadenitis, BCG diease, disseminated *M. avium* infection, multifocal osteomyelitis	Disseminated *M. Avium* infection	Scalp biopsy specimen grew *M. avium intracellulare*	Sharma VK, 2015
**13**	M	18 mo	Mexican	Vaccinated	c.819_822delTAAT	p.Asn274Hisfs*2	818del4, N274Hfs*2	Frameshift	Heterozygous	VI	AD-partial	NA	Femoral biopsy confirmed osteomyelitis, culture grew *Staphylococcus aureus*, axillary lymphadenopathy, hemophagocytic lymphohistiocytosis (HLH)		Nitro-blue tetrazolium reduction assay was normal	Staines-Boone AT, 2017
**14**	F	6 mo	Argentinian	Vaccinated	c.563_566del	p.Leu188Argfs*14	561del4, L188Rfs*14	Frameshift	Homozygous	V	AR-complete	+	Disseminated BCG infection, *M. avium* infection	Disseminated BCG and *M. avium* infection	BCG was identified on smear and culture	Rosenzweig S, 2002
**15**	F	5 y	Spanish	No	c.188T>G	p.Val63Gly	V63G	Missense	Homozygous	II	AR-partial	+	Disseminated infection with multifocal osteomyelitis due to *M. avium* complex and *M. szulgai*		TNF-α production was fivefold lower than in other family members, normal CD119 expression	Allende LM, 2001

**16**	M	15 mo	NA	No	c.805del	p.Tyr269Ilefs*8	805delT, Y269Ifs*8	Frameshift	Heterozygou s	VI	AD-partial	+++	Disseminated *M. intracellulare* infection multifocal osteomyelitis and pneumonia		*M. intracellulare* was isolated from bronchoalveolar lavage fluid, CD119 was highly expressed on monocytes by FC	Rose DM, 2014
**17**	F	7 y	Caucasian	NA	c.832G>T	p.Glu238*	832G>T	Frameshift	Heterozygou s	VI	AD-partial	+++	Osteomyelitis of the radius and femur, lytic and sclerotic lesions of the calcaneus, cultures grew *M. avium* complex		Overexpression of CD119 on T cells and monocytes, reduced STAT1 nuclear translocation after IFN-γ stimulation	Villella A, 2001
**18**	F	4 mo	Egyptian	Vaccinated	c.1424C>T	p.Ser475Phe	S485F	Missense	Homozygous	VII	AR	NA	BCGosis, generalized lymphadenopathy	Disseminated BCG infection	High plasma IFN-γ level	Galal N, 2012
**19**	F	5 mo	Chinese	Vaccinated	c.795_796insCT	p.Ile266Leufs*12		Frameshift	Heterozygou s	VI	AD-partial	+++	*M. avium* osteomyelitis	*M. avium* osteomyelitis	High cell-surface IFNGR1 expression by FC, low response to IFN-γ (pSTAT1 by FC)	Luo Q, 2023
**20**	F	20 y	Polish	Vaccinated	c.260T>C	p.Ile87Thr	I87T	Missense	Homozygous	III	AR-partial	NA	Disseminated *M. avium* infection, osteomyelitis	Disseminated M. avium infection	Blood samples showed impaired responses to IFN-γ. B BAAR in sputum.	Remiszewski P, 2006
**21**	M	12 mo	Mexican	Vaccinated	c.805del	p.Tyr269Ilefs*8	805delT, Y269Ifs*8	Frameshift	Heterozygou s	VI	AD-partial	+++	Lymphadenitis, BCG infection, mycobacterial liver infection, osteomyelitis, miliary tuberculosis, positive cultures for *M. bovis* and *Candida parapsilosis*		Poor STAT1 phosphorylation by FC and WB, CD119 overexpression.	**THIS REPORT**
**22-30**	5F, 4M	2-12 mo	Portuguese, Chilean	Vaccinated	c.260T>C	p.Ile87Thr	I87T	Missense	Homozygous	III	AR-partial	+	BCG-itis, disseminted salmonelosis, osteomyelitis, toxoplasmosis	BCG infection	Low IFNGR1 expression and reduced binding of IFN-γ to the receptor	Sologuren, 2011.
**31-48**	11F, 7M	6-36 mo	USA, Irish, German, Italian, Moroccan	Vaccinated or not	c.819_822delTAAT	p.Asn274Hisfs*2	818del4, N274Hfs*2	Frameshift	Heterozygou s	VI	AD-partial	+++	Disseminated BCG and *M. avium* infection with multifocal osteomyelitis in 12/18 patients	Disseminated BCG and *M. avium* infection	High cell-surface IFNGR1 expression by FC; low response to IFN-γ; no STAT1 nuclear translocation (EMSA)	Jouanguy E, 1999.
**49-51**	M	8 mo-3 y	Japanese	Vaccinated	c.819_822delTAAT	p.Asn274Hisfs*2	818del4, N274Hfs*2	Frameshift	Heterozygou s	VI	AD-partial	+++	Disseminated BCG infection, *M. avium* osteomyelitis	Multiple osteomyelitis due to BCG or *M. tuberculosis*	Higher IFN-γ receptor expression in patients compared with controls	Hoshina T, 2011
**52**	M	2 y	Japanese	Vaccinated	c.813_816del	p.Lys272Leufs*4	811del4	Frameshift	Heterozygou s	VI	AD-partial	+++	BCG multiple osteomyelitis, *M. bovis* (BCG Tokyo 172) from bone specimen	Multiple BCG osteomyelitis (non-recurrent)	IL-12 and IFN-γ production normal, IFNGR1 expression increased on monocytes (FC)	Obinata K, 2013
**53**	F	1 y	Japanese	Vaccinated	c.819_822delTAAT	p.Asn274Hisfs*2	818del4, N274Hfs*2	Frameshift	Heterozygou s	VI	AD-partial	+++	Multiple osteomyelitis due to	Recurrent multifocal	IL-12 and IFN-γ production normal,	Obinata K, 2013

													M. bovis (BCG Tokyo 172) from bone biopsy cultures, axillary lymphadenitis, skin eruptions/abscesses, recurrent course	osteomyelitis due to BCG	IFNGR1 expression increased on monocytes (FC)	

FC: flow cytometry. WB: Western Blot.
