# Supplementary material for: Mycobacterial infections and recurrent osteomyelitis in a patient with an autosomal dominant mutation in IFN-gamma receptor 1 with overexpression of the receptor
Source: Int J Infect Dis. Author manuscript; Available in PMC 2026 Nov 5. (PMC13739071; doi:10.1016/j.ijid.2026.109104)
Supplement: Supplementary file 14 [file NIHMS2218719-supplement-Supplementary_Table_S4.pdf]

Supplementary Table S4 Summary of distinct Human Genetic variations related to altered response to IFN-γ and their main clinical characteristics. Emphasis is placed on the frequency of osteomyelitis reported in cases of distinct IEI.

**Clinical Characteristics**	**AR *IFNGR1* (n=25)**	**AD *IFNGR1* (n=51)**	***STAT1* GOF (n=290)**	***STAT1* LOF (n=64)**	**AIGA excl. Zhao 2025 (n=13)*^**	**AIGA incl. Zhao 2025 (n=515)*^**
Mycobacterial disease	23/25 (92.0%)	44/51 (86.3%)	21/290 (7.2%)	57/64 (89.1%)	13/13 (100%)	431/515 (83.7%)
Disseminated mycobacterial infection	15/25 (60.0%)	39/51 (76.5%)	6/290 (2.1%)	29/64 (45.3%)	12/13 (92.3%)	438/515 (85.0%)
Lymph node infection	16/25 (64.0%)	31/51 (60.8%)	9/290 (3.1%)	23/64 (35.9%)	13/13 (100%)	433/515 (84.1%)
Lung infection	11/25 (44.0%)	12/51 (23.5%)	138/290 (47.6%)	10/64 (15.6%)	12/13 (92.3%)	316/515 (61.4%)
Osteomyelitis	11/25 (44.0%)	39/51 (76.5%)	4/290 (1.4%)	11/64 (17.2%)	13/13 (100%)	225/515 (43.7%)
Recurrent and/or multifocal osteomyelitis	8/25 (32.0%)	37/51 (72.5%)	NR	10/64 (15.6%)	12/13 (92.3%)	NR¬
Other bacterial infections (non-mycobacterial)	11/25 (44.0%)	4/51 (7.8%)	212/290 (73.1%)	8/64 (12.5%)	5/13 (38.5%)	107/515 (20.8%)
Fungal infections	3/25 (12.0%)	2/51 (3.9%)	283/290 (97.6%)†	7/64 (10.9%)	6/13 (46.2%)	166/515 (32.2%)
Viral infections	2/25 (8.0%)	2/51 (3.9%)	114/290 (39.3%)	21/64 (32.8%)	3/13 (23.1%)	69/515 (13.4%)

Abbreviations: AR, autosomal recessive; AD, autosomal dominant; GOF, gain-of-function; LOF, loss-of-function; IFN-γ, interferon gamma; AIGA, anti–interferon-gamma autoantibodies; NR, not reported.

* All patients with anti–IFN-γ autoantibodies (AIGA) reported to date are from Asia.
^ For AIGA, two values are shown for each infection category: the left value excludes the 502 cases reported by Zhao et al. (2025) (n=13), and the right value includes those cases (total n=515).
¬ In Zhao et al. (2025) (n=502), recurrent and/or multifocal osteomyelitis was not specifically reported; therefore, this item is marked as NR (not reported) in the “AIGA incl. Zhao 2025” column. When considering only the other reports (n=13), all 13 patients had osteomyelitis and 12 had recurrent and/or multifocal bone infection.
† In *STAT1* GOF, 269 of the 283 patients with fungal infections had mucocutaneous involvement.

References by category

AR *IFNGR1*: Sologuren 2011; Chantrain 2006; Jouanguy 1997; Jouanguy 2000; Taramasso 2014; Rosenzweig 2002; Galal 2012; Remiszewski 2006.
AD *IFNGR1*: Okada 2007; Staines-Boone 2017; Dorman 2004; Rose 2014; Villella 2001; Luo 2023; Jouanguy 1999; Obinata 2013; Hoshina 2011; **This report**
***STAT1* GOF:** Toubiana 2016; Pedraza-Sanchez 2017; Leiding 2018
***STAT1* LOF**: Liu 2022; Hirata 2013; Ye 2022; Tsumura 2022; Ueki 2017; Kagawa 2017; Lim 2025.

AIGA*:* Tang 2021; Nishimura 2015; Tang 2023; Zhao 2025.
